# Sustainable Polyaddition Path to Polyesters via Catalytic Homocoupling of Renewable Dicrotonate Monomers

**DOI:** 10.1021/acs.macromol.5c02308

**Published:** 2025-11-30

**Authors:** Asif Shabbir, Mahsa Saeidi, Braden D. Pickle, Nicholas J. Tabbah, Michael L. McGraw

**Affiliations:** a Fulbright College of Arts and Science, Department of Chemistry & Biochemistry, 3341 University of Arkansas, 345 North Campus Walk, Fayetteville, Arkansas 72701, United States; b Graduate School and International Education, Department of Materials Science & Engineering, University of Arkansas, 340 Norht Campus Walk, Fayetteville, Arkansas 72701, United States

## Abstract

The development of biorenewable polyhydroxyalkanoate (PHA) chemistry has generated interest in crotonate-based monomers as versatile building blocks. Crotonates can be obtained through the pyrolysis of PHAs, offering both a sustainable end-of-life pathway for PHAs and a renewable route to crotonate monomers. This study presents a new polyaddition-type step-growth polymerization of dicrotonates in which alkene-functionalized polyesters are synthesized using potassium *tert*-butoxide as a simple base catalyst. The reaction proceeds rapidly at room temperature, achieving >99% conversion within seconds and requiring no byproduct removal. The properties of the resulting polyesters can be tuned via the choice of bridging diol, and the materials are degradable by hydrolysis. The polymers reported herein are soft, amorphous solids with predominantly cyclic architectures under standard conditions, while linear analogs can be accessed through a chain-end-capping strategy. The work presented herein bridges key principles of polymer sustainability—including biorenewability of feedstocks, energy-efficient synthesis, and degradable end-of-life pathways—with practical considerations essential for real-world application, such as operational simplicity, scalability, cost-effectiveness, and supply chain accessibility.

## Graphical abstract

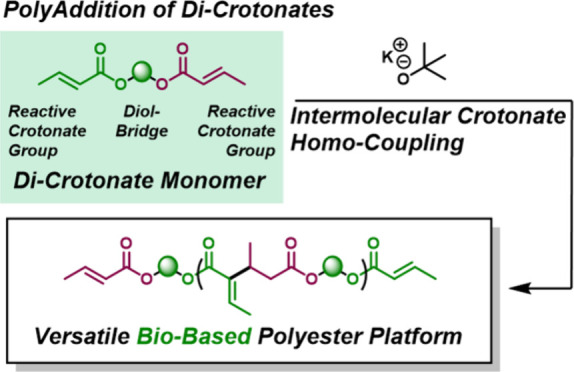

## Introduction

In recent years, the global polymer pollution crisis has intensified with plastic production reaching unprecedented levels of more than 400 million metric tons per year.^1–5^ The vast majority of these materials—such as polyethylene, polypropylene, polystyrene, and polyvinyl chloride—are polyolefins composed of carbon–carbon backbones, rendering them resistant to both recycling and biodegradation within practical timeframes.^6–10^ Consequently, there is an urgent need to replace such materials with more sustainable alternatives, such as polyesters,^11–19^ which contain carbon–oxygen bonds in the backbone^20^ that enable chemical recycling^21–27^ and environmental degradation.^28–33^

In this context, polyhydroxyalkanoates (PHAs)^32–44^ have gained significant attention, with hundreds of publications each year reporting innovations in their synthesis, properties, and applications. PHAs are notable for being primarily derived from renewable carbon sources—often via microbial fermentation of waste streams—and are enzymatically biodegradable over relatively short time scales, mitigating their environmental persistence. These materials can be synthesized either biologically^36^ or chemically,^44,45^ offering a broad range of structural and functional diversity.

Despite these advantages, PHAs lack a robust postconsumer end-of-life strategy that enables recovery of the embedded carbon and energy.^46–49^ Chemical recycling approaches often lead to the formation of a thermodynamically stable cyclic trimer, which is difficult to repolymerize.^50–53^ However, pyrolytic decomposition of PHAs has been shown to selectively yield crotonic acid (CA),^54–58^ a highly reactive small molecule that could serve as a valuable intermediate in a sustainable chemical supply chain (Figure 1). Yet, despite its renewable origin and potential utility, crotonate chemistry remains underexplored and underutilized. At present, there are few, if any, practical downstream applications for CA, leaving a critical opportunity gap in the valorization of PHA-derived products. Thus, the development of a useful application for the crotonate unit would not only open a new and sustainable area of chemistry but also serve as a key enabling force for the broader adoption and success of existing PHA technologies.

**Figure 1:**
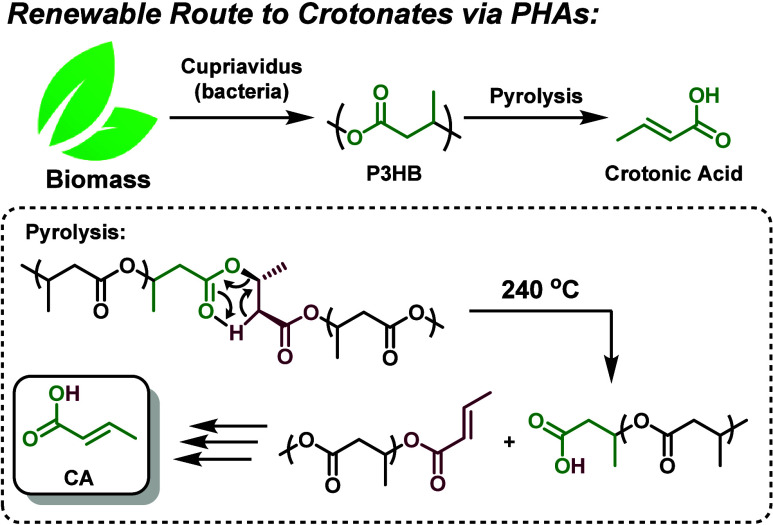
Renewable route to CA via pyrolysis of biopolymer poly­(3-hydroxybutyric acid) (P3HB).

Several crotonate-based polymerization strategies have emerged in recent years. Notably, the chain-addition polymerization of crotonates, first reported by Hatada et al. and Ute et al.^59,60^ and later by Chen and McGraw^61,62^ and further developed by Abe et al.,^63–65^ Hong et al.,^66^ and others, has demonstrated potential utility in producing functional materials. However, these polymerizations ultimately yield poly­(crotonates) with carbon–carbon backbones, rendering them chemically analogous to poly­(methacrylates) and similarly resistant to degradation. As such, while these materials may be useful in select contexts, they offer limited advantages from a sustainability standpoint other than biorenewability. Very recently, Sardon et al.^67^ reported a degradable crotonate-based polyester synthesized via an elegant alternating copolymerization strategy. By employing 2-methylene-1,3-dioxepane as a comonomer and leveraging the crotonate inability to homopolymerize under free radical conditions, they achieved controlled radical copolymerization.

In contrast, Waymouth and co-workers reported in 2015 a catalytic dimerization (homocoupling) of crotonate (Scheme 1), representing a compelling strategy to upgrade the C_4_ crotonate unit into C_8_ diesters.^68^ Waymouth reported both a basic vinylogous Michael addition reaction (basic pathway, Scheme 1) and a nucleophilic Rauhut–Currier type pathway when N-heterocyclic carbenes were used (nucleophilic pathway). These dimers possess ester linkages that are amenable to polycondensation with diols, offering a route to degradable polyesters.

**Scheme 1:**
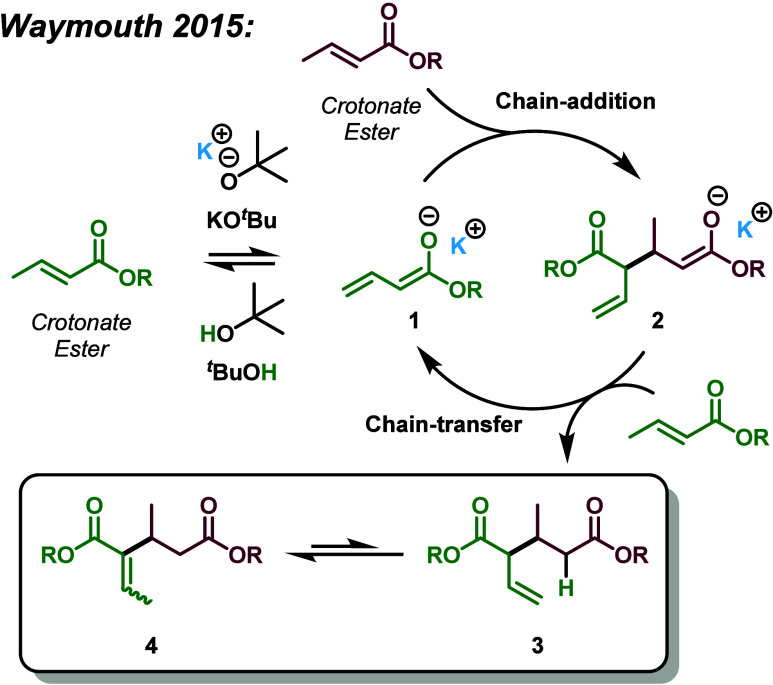
Base-Catalyzed Dimerization of Crotonates, Specifically, the Basic Vinylogous Michael Addition Pathway (as Opposed to the Nucleophilic Rauhut–Currier Pathway)

The base-catalyzed pathway begins with deprotonation of a crotonate ester at the γ-position by a strong base such as potassium *tert*-butoxide (KO*^t^* Bu), generating enolate species **1**. This nucleophilic enolate undergoes a conjugate addition to a second crotonate ester, yielding dimeric enolate intermediate **2**. Chain transfer from **2** can proceed via several pathways: (a) deprotonation of another crotonate ester; (b) deprotonation of a neutral dimeric product such as **3** or **4**, leading to a conjugated dimeric enolate (not shown); (c) deprotonation of the conjugate acid, *tert*-butanol (*^t^* BuOH); or (d) via an intramolecular 1,3-proton transfer followed by one of the above steps. Notably, under optimized conditions, further chain addition beyond the dimer stage—i.e., attack of enolate **2** on a third crotonate ester—is not observed. In Waymouth’s original report, only the thermodynamic product **4** was isolated, with an observed ∼80:20 E/Z ratio; the kinetic product **3** was not detected, though its transient formation remains postulated.

Building on this insight, we proposed an alternative approach to step-growth polymerization of bis-functional crotonate esters—herein referred to as dicrotonates (DCs). These DCs could then undergo base-catalyzed homocoupling, akin to Waymouth’s dimerization, to facilitate a step-growth polyaddition (Scheme 2). The general concept, in the context of DC monomers, is that a simple Bro̷nsted base can deprotonate a crotonate γ-proton—from either a DC monomer or a growing poly­(DC) chain (**5**)—to form the crotonoyl dienolate **6**. This enolate **6** can then undergo chain addition to another crotonate group, resulting in the formation of a new C—C bond and an increase in the chain length (Scheme 2). However, unlike traditional chain-growth polymerization, which is characterized by consecutive chain additions, the enolate product **7** formed after chain addition is immediately protonated (either by the conjugate acid *^t^* BuOH or by another crotonate group), yielding a stable repeat unit. Notably, due to the difunctional nature of the monomer, active crotonate groups remain at the chain ends, allowing for continuous turnovers of this process, thereby leading to an overall step-growth mechanism. This strategy leverages the thermodynamic driving force of C—C bond formation akin to chain growth polymerizations, while harnessing the intrinsic sustainability advantages of polyaddition, offering a simple and efficient path to degradable, bioderived polyesters.

**Scheme 2:**
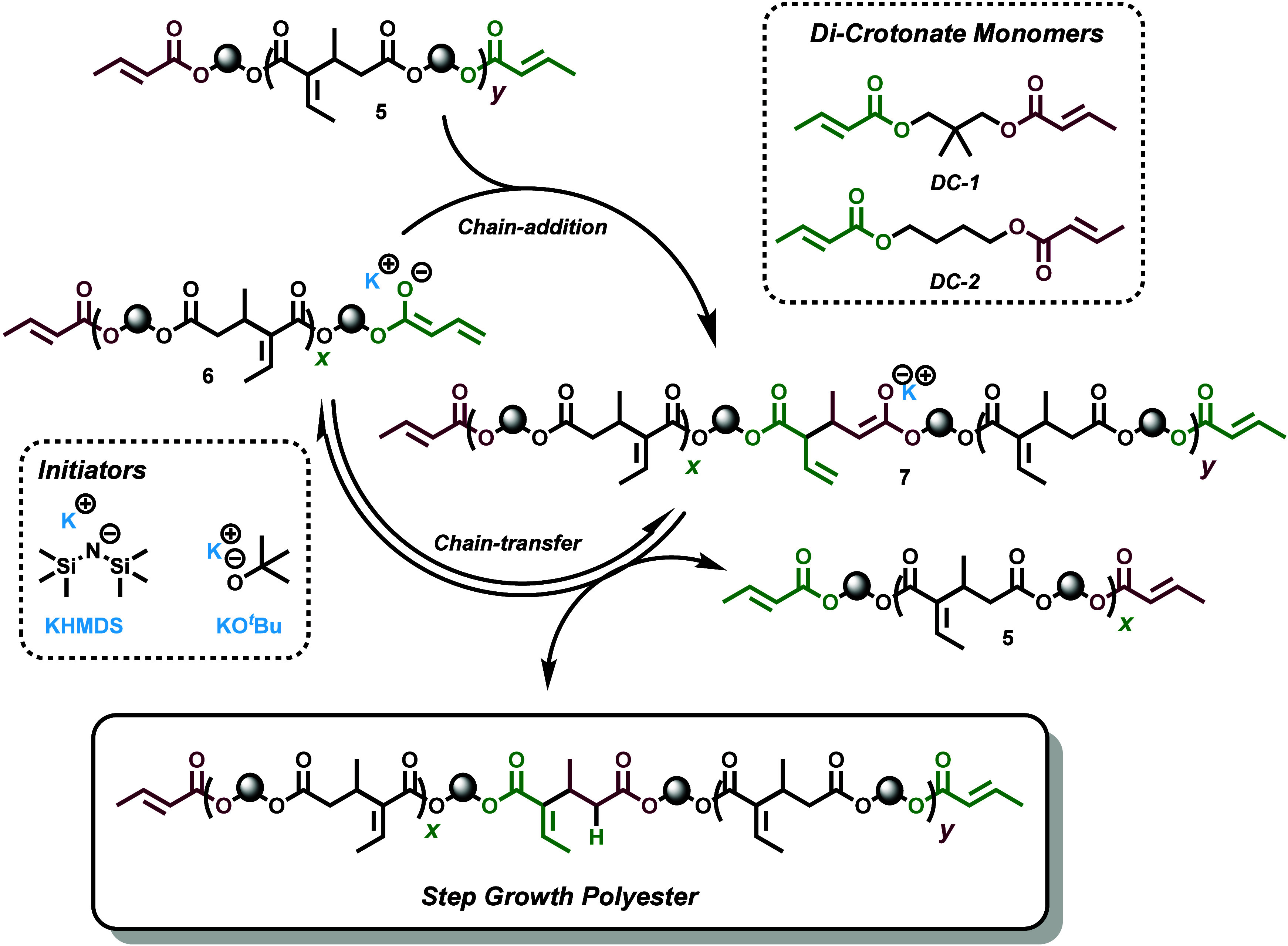
Polyaddition of DCs by Base-Catalyzed Intermolecular Crotonate Coupling

This polyaddition strategy is particularly compelling from both polymer chemistry and sustainability perspectives. Unlike polycondensation, polyaddition proceeds via direct bond formation without the release of a leaving group, thereby eliminating the need for byproduct removal during polymerization. This feature enables polymerization to occur “in the mold” under ambient conditions, without heating or an elaborate setup. The reaction is spontaneous at room temperature (RT) and proceeds rapidly—achieving >99% conversion within seconds—a crucial advantage for step-growth processes, where high conversion is necessary to reach high number-average molecular weight (*M*_n_). In contrast to polyurethanes, the other major class of industrial polyaddition polymers, this reaction avoids the use of toxic or otherwise privileged functional groups such as isocyanates. Moreover, because the process is a homocoupling reaction rather than a traditional A–B step-growth system, it is not constrained by the precise stoichiometric balance limitation dictated by the Carothers equation, offering greater robustness in practical applications. Importantly, the platform is structurally tunable via modification of the bridging diol unit. This allows for incorporation of various biorenewable diols—such as neopentyl glycol or 1,4-butanediol, as used in this study—while maintaining the degradable polyester backbone. The resulting polymers exhibit a spectrum of thermomechanical properties, supporting a versatile and sustainable materials platform. Finally, we anticipate these polymers to be biodegradable, owing to their polyester backbone and structural similarity to PHAs, which may promote enzymatic degradation.

While developing this platform, we became aware that Lu^69^ and co-workers had independently arrived at a similar concept, publishing the polyaddition of DCs in 2024. Their work employed *N*,*N*′-bis­(imidazolyl) guanidinylphosphine catalysts to promote the Rauhut–Currier-type coupling of DCs. This elegant study provided important insights into the nucleophilic pathway for crotonate homocoupling, particularly with respect to stereochemical control. Lu’s team also explored a broad scope of DCs, demonstrating access to diverse polymers with tunable thermomechanical properties. Additionally, they highlighted the potential of the resulting in-chain alkenes for postpolymerization functionalization via thiol–ene click chemistry.

In contrast, our work presents a more practical and accessible route to poly­(DC), leveraging a simplified base-catalyzed pathway using commercially available KO*^t^* Bu. Our approach emphasizes operational simplicity, cost-effectiveness, and sustainability by favoring more concentrated conditions and avoiding the use of inconvenient solvents, such as dimethylformamide. Furthermore, this study provides new mechanistic insights into the polyaddition process, including evidence for macrocyclization, chain branching, and end-group capping events—factors that are critical for understanding and controlling the molecular architecture of these materials. Herein are the results of our investigation of the polyaddition of DCs.

## Results and Discussion

### 1. Preparation of DC Monomers

The synthesis of neopentyl glycol and 1,4-butanediol bridged DCs (**DC-1** and **DC-2**, respectively, Scheme 2) was trivial and involved the one step condensation of the corresponding alcohols with crotonic acid in toluene at reflux, while using a Dean–Stark trap to remove water. Both DCs were purified through distillation or flash chromatography (Figures S24 and S25), obtained at high yields (96% and 88%, respectively), and characterized by mass spectrometry (MS) (Figures S15 and S16), ^1^H NMR (Figures S1 and S4), and ^13^C NMR (Figures S3 and S6). Both DCs were chosen for their simplicity and biorenewability. The neopentyl glycol bridge found in DC-1 was chosen specifically to offer more steric hindrance to the carbonyls and to eliminate the possibility of base-catalyzed elimination reactions as this bridge does not contain protons β to the carboxylate leaving group.

### 2. Polymerization of DCs: Initial Runs and Observations

Next, polymerization reactions were carried out according to the following conditions. THF was the solvent chosen as it is the best solvent for monocrotonate dimerization reactions.^68^ All runs were carried out at RT; however, temperatures up to ∼60 °C were briefly observed due to polymerization exotherm. Two bases, KO*^t^* Bu and potassium bis­(trimethylsilyl)­amide (KHMDS) were chosen for their simplicity and commercial availability. Both bases were applied as 1.0 M stock solutions (in THF) and injected into a stirring mixture of DC and THF. Importantly, the terminal alkene shown in intermediate **7** (Schemes 2–4) is inferred and was never directly observed. Instead, we observe the conjugated alkene represented in the “step-growth polyester” (Schemes 2–4), which is the thermodynamic product following base-catalyzed rearrangement. This conjugated alkene was observed at an ∼80:20 E:Z ratio in all cases. Conversions were measured by ^1^H NMR by comparing twice the sum of the integrals of both product E/Z isomers to the integral of the crotonate α-alkene peak (since 2 crotonate alkenes are consumed in the formation of 1 product alkene). For a more descriptive example, see Figure S9. Tables 1 and 2 show the results of the polyaddition of DC-1 and DC-2 under varying timeframes, catalyst loadings, and catalysts.

**Table 1:**
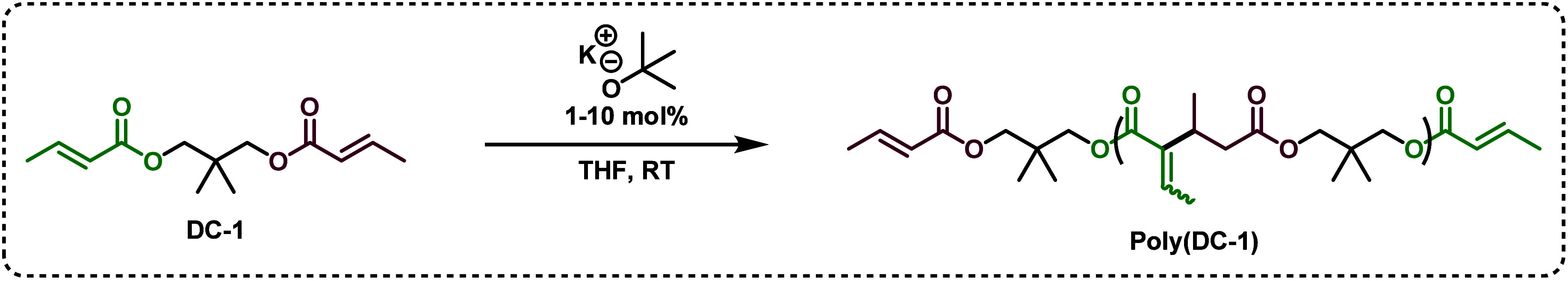
Polymerization Runs for **DC-1** ^a^

run no.	KO^*t*^Bu mol %	time	conv. (%)^b^	*M*_n_ (kg/mol)^c^	*Đ* ^c^
1	10	5 min	>99	9.12	2.07
2	10	60 min	>99	9.13	2.07
3	10	6 h	>99	8.44	2.44
4	1	5 min	59	1.22	1.37
5	5	5 min	98	10.6	2.66
6	10	5 min	>99	9.12	2.07
7	20	5 min	>99	6.19	1.56
8	10^d^(KHMDS)	5 min	>99	9.16	2.30

a Conditions: each reaction contains 0.25 mL of **DC-1**, and [DC-1]_0_ = 1 M in THF; temperature = 25 °C. Polymerizations initiated by initiated by injecting KO*^t^* Bu solution premixed in THF.

b Conversion determined by H NMR.

c Number-average molecular weight (*M*_n_) and dispersity (*Đ*) determined by GPC at 25 °C in THF coupled with a DAWN multi(8)-angle light scattering detector and an Optilab dRI detector for absolute molecular weights. The d*n*/d*c* value of poly­(DC-1) was determined to be 0.0729 mL/g in THF (Figure S27).

d KHMDS was used as the base initiator instead of KO*^t^* Bu.

**Table 2:**
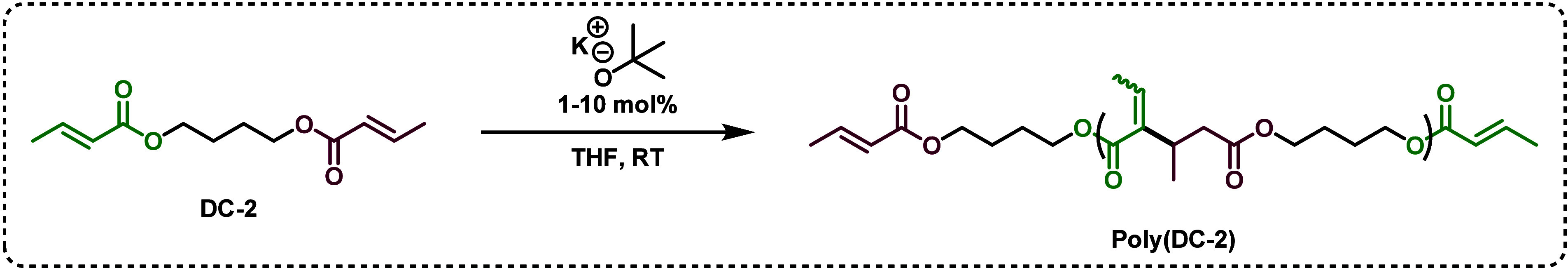
Polymerization Runs for **DC-2** ^a^

run no.	KO^*t*^Bu mol %	time	conv. (%)	*M*_n_ (kg/mol)	*Đ*
9	10	5 min	>99	14.0	4.20
10	10	60 min	>99	13.5	4.19
11	10	6 h	>99	14.5	3.90
12	1	5 min	79	2.83	1.58
13	5	5 min	97	15.2	7.32
14	10	5 min	>99	14.0	4.20
15	20	5 min	>99	7.61	1.80
16	10 (KHMDS)	5 min	>99	13.8	4.00

a Conditions: each reaction contains 0.25 mL of **DC-2**, and [DC-2]_0_ = 1 M in THF; temperature = 25 °C. Polymerizations initiated by initiated by injecting KO*^t^* Bu solution premixed in THF. The d*n*/d*c* value of poly­(DC-2) was determined to be 0.0647 mL/g in THF (Figure S28).

**Scheme 3:**
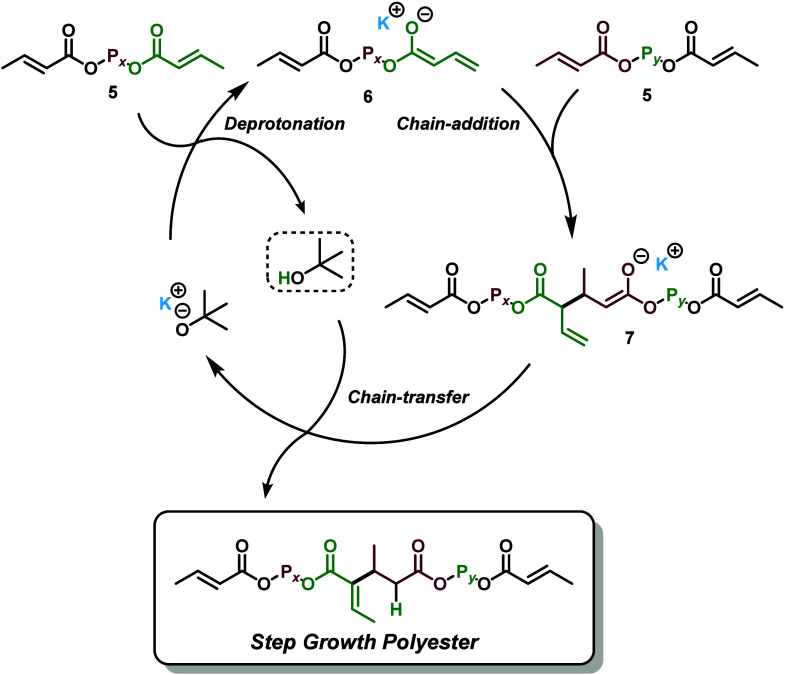
Polyaddition of DCs by KO*^t^* Bu Highlighting the Role of Conjugate Acid *^t^* BuOH in Facilitating Chain Transfer via Protonation of Reactive Enolate **7** ^a^

a Note that polymers **5**, **6**, and **7** shown here in a more condensed representation, are analogous to the same polymers **5**, **6**, and **7** shown in Scheme 2.

**Scheme 4:**
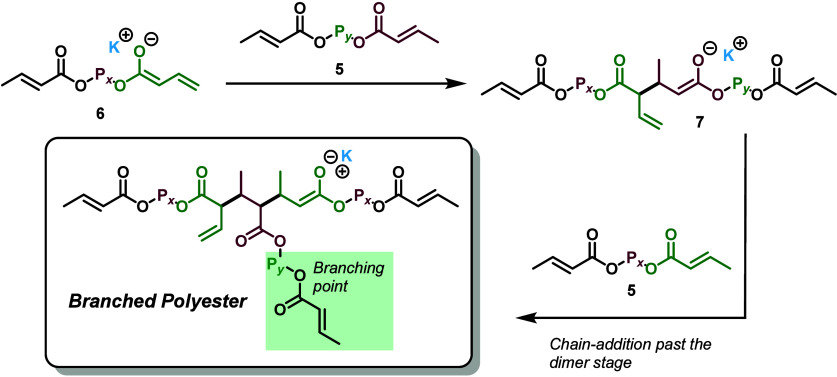
Chain Addition Past the Dimer Stage, Which Results in Cross-Linking “Branch Points” If Chain Addition Is Competitive with Chain Transfer

The most notable observation from these initial experiments is that reaction time had minimal impact on conversion and no positive effect on *M*_n_. The reaction appears to be highly efficient, reaching near-complete conversion (>99%, runs 1 and 9) within just 5 min, with no measurable increase in conversion observed after 60 min (>99%, runs 2 and 10). Reducing KO*^t^* Bu loading to 1 mol % led to incomplete conversion (runs 4 and 12), likely due to reversible deprotonation of acidic sites within the poly­(DC) backbone by **6**, resulting in catalyst sequestration late in the reaction, or termination via Claisen condensation (*vide infra*). A minimum KO*^t^* Bu loading of 5 mol % was required to achieve ≥97% conversion (runs 5 and 13). For the remainder of the study, a 10 mol % catalyst loading was used to ensure >99% conversion. Highly basic KHMDS operated similarly under the conditions utilized in Tables 1 and 2 (runs 8 and 16) and gave similar results in terms of conversion and *M*_n_. However, at higher scales or at higher [DC]_0_, KHMDS proved problematic due to its promotion of cross-linking through extending the lifetime of intermediate **7** ([Fig sch3] and 4). Therefore, KO*^t^* Bu was used for the remainder of the study. The poly­(DC-1) and poly­(DC-2) products were characterized by ^1^H NMR (Figures S7 and S12, respectively), and ^13^C NMR (Figures S11 and S14, respectively).

While KO*^t^* Bu and KHMDS are very similar in that they will both produce the same potassium enolate **6** (Scheme 2 and 3), the conjugate acid of KHMDS, hexamethyldisilazane, is much more inert and less likely to participate subsequent to the initial deprotonation, whereas the conjugate acid of KO*^t^* Bu (*^t^* BuOH) is much more acidic and capable of acting as a chain transfer agent (Scheme 3).

We found the acidity of the conjugate acid to be extremely relevant, as the polymers generated via the KHMDS catalyst were typically insoluble, especially at higher concentrations/scales. We hypothesize that this is due to chain addition past the dimer stage competing with chain transfer steps, which results in branched polyesters (Scheme 4). Branched polyesters with 3 or more reactive crotonate groups will eventually construct large, cross-linked macromolecules that become insoluble. Thus, it would seem that the conjugate acid *^t^* BuOH is extremely important for the chain transfer step and preventing continued chain addition past the dimer stage. In fact, excess *^t^* BuOH can be used to ensure a highly protic environment where enolate **7** is quenched immediately following its generation to suppress chain branching under harsher, more concentrated conditions (*vide infra*, Table 3).

**Table 3:** Polymerization Runs^a^ of **DC-1** and **DC-2** at Variable Concentrations

run no.	DC-X	[DC-X]_0_ (M)	[^*t*^BuOH] (M)^b^	conv. (%)	*M*_n_ (kg/mol)	*Đ*
17	DC-1	1.0	0	>99	10.3	2.76
18	DC-1	1.5	0	>99	13.8	6.59
19	DC-1	2.0	0	>99	24.9^c^	14.9
20	DC-1	2.5	0	97	18.7^c^	13.5
21	DC-1	1.0	1.0	>99	9.11	1.79
22	DC-1	1.5	1.5	>99	10.9	2.21
23	DC-1	2.0	2.0	98	11.9	2.82
24	DC-1	2.5	2.5	98	13.3	5.16
25	DC-2	1.0	0	>99	15.9	5.07
26	DC-2	1.5	0	98	^d^	
27	DC-2	2.0	0	97		
28	DC-2	2.5	0	97		
29	DC-2	1.0	1.0	99	9.91	1.89
30	DC-2	1.5	1.5	98	11.5	2.45
31	DC-2	2.0	2.0	99	12.0	3.12
32	DC-2	2.5	2.5	98	13.7	4.65

a Conditions: each reaction contains 0.25 mL of **DC-1** or **DC-2**. [DC-1; DC-2]_0_/[KO*^t^* Bu]_0_ = 10/1. Time = 5 min. Solvent = THF and in some runs.

b *^t^* BuOH was used as a cosolvent and proton transfer agent, always at the same conc as DC-X.

c *M*_n_ and *Đ* of the soluble fraction, as these runs produce a networked gel polymer.

d No soluble fraction recovered as reaction was totally networked.

### 3. Mechanistic Investigation into the Polyaddition of DCs

To verify true step-growth behavior, we obtained a *M*_n_ versus conversion profile for the polymerization of **DC-1** under the conditions used for run 1. However, because this polymerization reaches >97% conversion within the first few seconds under these conditions, it is difficult to obtain aliquots at different conversion points naturally. Instead, we obtained these data by adding the KO*^t^* Bu solution dropwise and collecting an aliquot after each drop of catalyst added. In other words, the incremental addition of catalyst leads to a corresponding incremental increase in conversion, after which the catalyst species is either terminated or sequestered by acidic protons in the product, enabling us to take samples at different conversion points. After determining the conversion and molecular weight (MW) data for several aliquots, the data was plotted as *M*_n_ vs conversion (Figure 2), by which we can reasonably infer that these polymerizations indeed proceed via a step-growth mechanism as supported by the Carothers-type increase in *M*_n_ per conversion.

**Figure 2:**
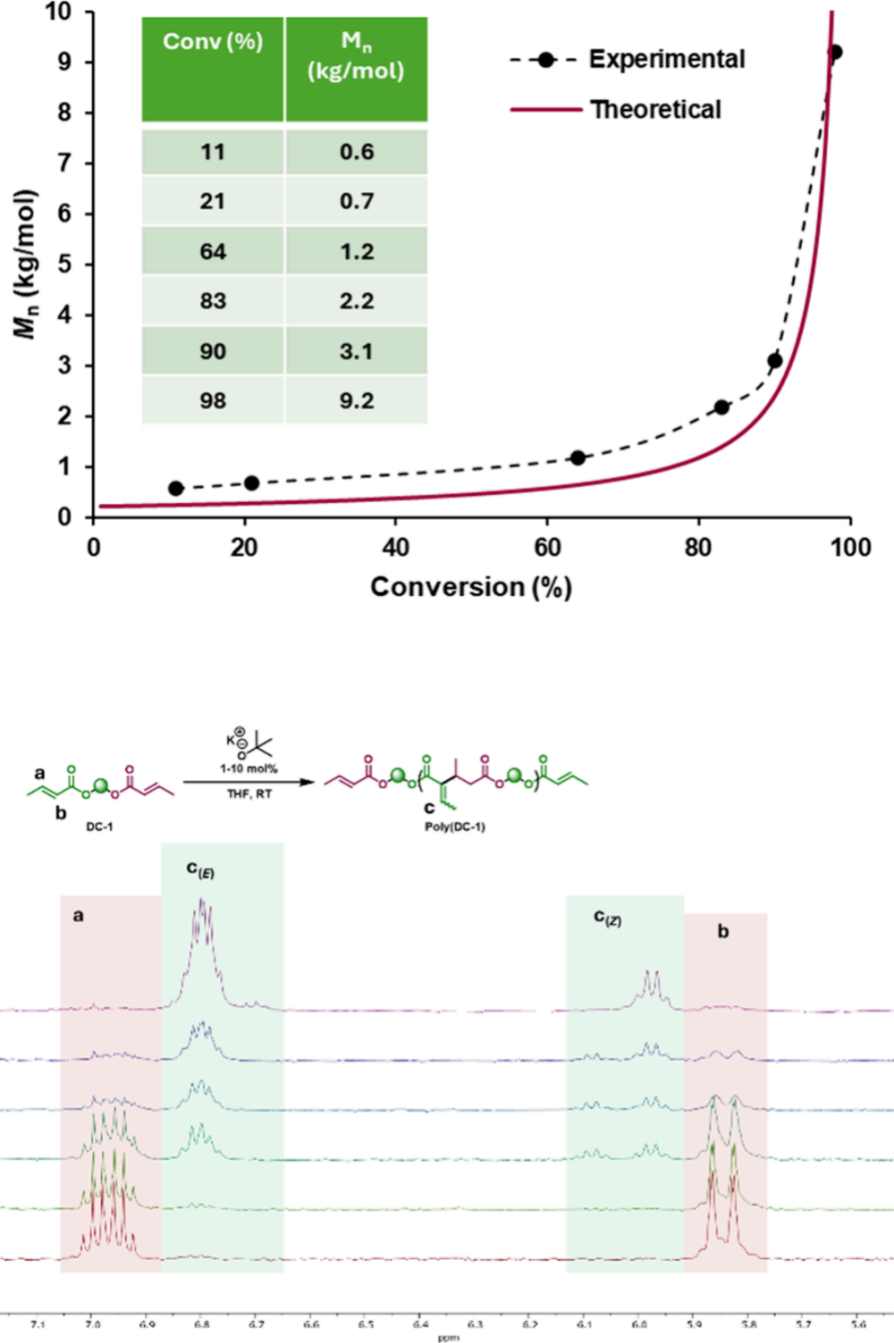
(Top) *M*_n_ vs conversion profile for **DC-1** polymerization (1.0 M in THF, 10 mol % KO^t^Bu), consistent with the theoretical Carothers-type *M*_n_ = *M*_0_ ÷ (1 – *p*) step-growth behavior. Inset: table of measured *M*_n_ and conversion values. (Bottom) Stacked ^1^H NMR spectra (alkene region) showing crotonate alkene protons a and b and product alkene protons c (E/Z isomers), for each aliquot in the plot/table. Spectra are ordered from the bottom (lowest conversion) to the top (highest conversion).

Importantly, the theoretical *M*_n_ for DC-1 at 99% conversion is 24 kg/mol. However, the polymers obtained from the polymerization of **DC-1** generally exhibit *M*_n_ values closer to ∼9 kg/mol (Table 1, runs 1–3). One potential explanation for this discrepancy is chain degradation. This hypothesis is partially supported by runs 7 and 15, where a higher catalyst loading of KO*^t^* Bu (20 mol % vs 10 mol %) was used. However, in both 20 mol % runs, the dispersity (*Đ*) decreased relative to their 10 mol % counterparts (run 6: *Đ* = 2.07, run 7: *Đ* = 1.56; run 14: *Đ* = 4.20, run 15: *Đ* = 1.80). This trend suggests that the lower-than-expected *M*_n_ values are not primarily due to random chain degradation, which would be expected to increase *Đ* rather than reduce it. Additionally, polymer degradation is not supported by MS analysis (*vide infra*).

A more plausible explanation for the lower-than-expected *M*_n_ values is intramolecular chain addition between the α- and ω-terminal crotonate groups, resulting in macrocyclization (Scheme 5).

**Scheme 5:**
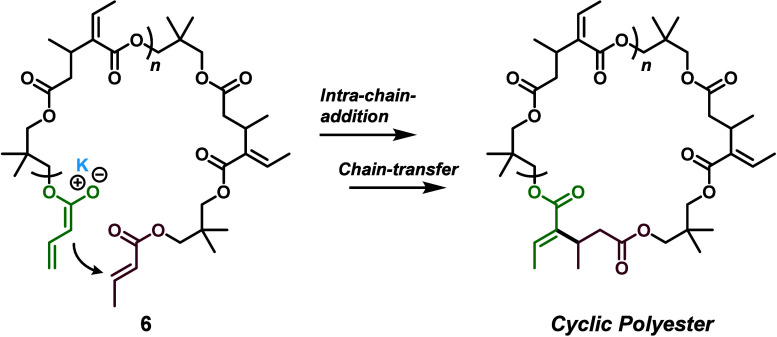
Intramolecular Chain Addition, Which Results in Cyclic polyesters

As there is no compelling reason to dismiss this possibility, we designed a simple kinetic experiment to probe the cyclization behavior. The rate of intermolecular chain addition (Scheme 2), which is the principle elementary step responsible for step-growth, should follow:

$${\mathrm{rate}}_{\mathrm{inter}}=k_{p}[5{]}_{t}[6{]}_{t}\;\alpha \;k_{p}[\mathrm{DC}{]}_{0}[{\mathrm{KO}}^{t}\mathrm{Bu}{]}_{0}$$

Here, we assume that [**5**]*_t_* is related to [DC]_0_ while [**6**]*_t_* is related to [KO*^t^* Bu]_0_. Similarly, the rate of intramolecular chain addition (cyclization, Scheme 5) is expected to follow:

$${\mathrm{rate}}_{\mathrm{intra}}=k_{c}[6{]}_{t}\;\alpha \;k_{c}[{\mathrm{KO}}^{t}\mathrm{Bu}{]}_{0}$$

Thus, reducing solvent volume (i.e., increasing [DC]_0_ and [KO*^t^* Bu]_0_) enhances the rate of intermolecular chain addition quadratically, while the rate of cyclization increases only linearly. Since intermolecular chain addition increases *M*_n_ while cyclization limits *M*_n_, the following proportion can be invoked:

$$M_{n}\propto \frac{k_{p}[5{]}_{t}[6{]}_{t}}{k_{c}[6{]}_{t}}\;∴\;\frac{k_{p}[5{]}_{t}}{k_{c}}\propto \frac{k_{p}[\mathrm{DC}{]}_{0}}{k_{c}}$$

Since [**6**]*_t_* (and by extension [KO*^t^* Bu]_0_) affects both processes (inter- and intramolecular chain addition) equally, it can be neglected (canceled out), and we see that increasing [DC]_0_ should result in increasing *M*_n_. This relationship allows *M*_n_ to serve as an indirect probe for the relative rates of propagation and cyclization as a function of [DC]_0_. Thus, if an increasing trend in *M*_n_ is observable vs increasing [DC]_0_, that is one line of indirect evidence that points toward cyclization. We therefore ran a set of experiments using [DC-1]_0_ = {1.0, 1.5, 2.0, 2.5 M}, the results of which can be seen in Table 3.

Polymerization of DC-1 at elevated concentrations (runs 18–20) generated significant exotherms, leading to solvent (THF) boiling. At 1.5 M (run 18), the product remained soluble; however, the 2.0 and 2.5 M runs (runs 19 and 20) yielded insoluble network polymers, likely due to uncontrolled chain addition beyond the dimer stage (Scheme 4). Soluble fractions isolated from these higher concentration runs exhibited substantially increased *M*_n_ values (24.9 and 18.7 kg/mol) and very broad *Đ* (*Đ* = 14.9 and 13.5, respectively). To suppress excessive chain branching, we introduced 1 equiv of *^t^* BuOH relative to [DC]_0_ to rapidly quench enolate **7** (Scheme 3) and prevent further chain addition (Scheme 4). This approach was effective as no further network formation was observed in runs 21–24 and dissolved polymer could easily be pushed through a 0.45 μm syringe filter. As expected, DC-1 showed increasing *M*_n_ and *Đ* with increasing concentration (runs 21–24). **DC-2** followed a similar trend (runs 25–28). The exceptionally high *Đ* values at higher concentrations suggest the coexistence of multiple, competing mechanistic pathways governing *M*_n_. One interpretation is that extensive macrocyclization occurs at low degrees of polymerization (DP), lowering the overall *M*_n_. However, chains that avoid early cyclization and surpass a critical DP threshold can continue growing to exceptionally high MWs, thereby increasing the weight-average molecular weight (*M*_w_). For example, in run 25, *M*_n_ = 15.9 kg/mol while *M*_w_ = 80.6 kg/mol.

This broadening of *Đ* with respect to concentration can be visualized and further interpreted by analysis of the GPC chromatograms, found in Figure S23A,C (**DC-1**) and Figure S23B,D (**DC-2**). The light scattering (LS) traces, which weight strongly toward larger particles, show a pronounced shift toward lower elution times for the 2.5 M samples, indicating a higher overall molecular weight population. In contrast, the differential refractive index (dRI) traces, which reflect total concentration, reveal that the 1.0 and 2.5 M samples have nearly identical low-MW distributions (≥22 min retention time) but differ significantly on the high-MW side (<22 min), with the 2.5 M sample skewed toward higher MW. This suggests that while the 2.5 M samples have populations with higher MW on average than the 1.0 M samples, this MW increase is only borne out in the high-MW fraction (i.e., the left side of the dRI bell-curve), while the low-MW fraction (right side of the dRI bell-curve) is largely the same in both cases.

The GPC data indicate that low-MW species are produced in both 1.0 and 2.5 M [DC]_0_, suggesting that the pathway responsible—most plausibly early stage macrocyclization—operates with similar efficiency regardless of concentration. In contrast, the high-MW fraction is markedly enriched at 2.5 M, as evidenced by the left-shifted LS traces and divergence in the high-MW side of the dRI curves. This pattern is consistent with a subset of chains in the more concentrated reactions escaping early cyclization and continuing to grow to a much higher MW, thereby disproportionately increasing *M*_w_ and *Đ*.

An alternative explanation is that slight branching in the more concentrated runs could raise the molecular weight without rendering the polymer insoluble, consistent with the observed *M*_n_–concentration correlation. While the cyclization pathway is our favored explanation based on consistency with other lines of evidence, the mechanism remains speculative at this stage.

Important to note is that this observed MW ceiling is consistent with Lu’s findings,^69^ where similar DCs plateaued at comparable *M*_n_ values via the Rauhut–Currier mechanism, despite reaching >99% conversion. It also aligns with Chen’s earlier work on umpolung coupling of dimethacrylates,^70^ which similarly plateaued around 10 kg/mol at full conversion.

Although increasing monomer concentration appears to promote higher *M*_n_, maintaining homogeneity becomes challenging above 2.5 M. Even with excess *^t^* BuOH, local hotspots can form, leading to thermal runaway, uncontrolled chain addition, and ultimately polymer networking and gelation. We anticipate that more sophisticated catalytic systems may enable the controlled polymerization of DC monomers under solvent-free conditions, offering a pathway to higher *M*_n_ materials. These efforts are currently under investigation.

To further investigate polymer structure and reaction mechanism, we performed matrix-assisted laser desorption/ionization time-of-flight (MALDI-TOF) MS on polymers from run 1 (poly­(DC-1), Table 1). The resulting spectrum is shown in Figure 3. The dominant series of peaks corresponds to the expected polymer structure **I**. It should be noted that the linear structure **I** depicted in Figure 3 may in fact correspond to the cyclic polymer (Scheme 5), as MALDI-TOF cannot distinguish between linear and cyclic architectures due to their identical mass profiles. Additionally, less intense sets of peaks were observed corresponding to Claisen condensation products, including monocondensed (**II**) and dicondensed (**III**) species, along with the associated mono- and diol leaving groups (**IV** and **V**), which appeared at relatively similar intensities. Notably, no peaks were detected that matched KO*^t^* Bu-mediated transesterification products—either from linear (theoretical end-group *m*/*z* = 165) or cyclic polyesters (theoretical end-group *m*/*z* = 97). This suggests that bulky KO*^t^* Bu does not attack ester carbonyls under the conditions used and, therefore, does not contribute to degradation via transesterification. Similarly, no signals were observed for hydrolyzed products of linear (theoretical end-group *m*/*z* = 109) or cyclic (theoretical end-group *m*/*z* = 41) poly­(DC-1), indicating that hydrolysis is also not operative under these (dry) conditions.

**Figure 3:**
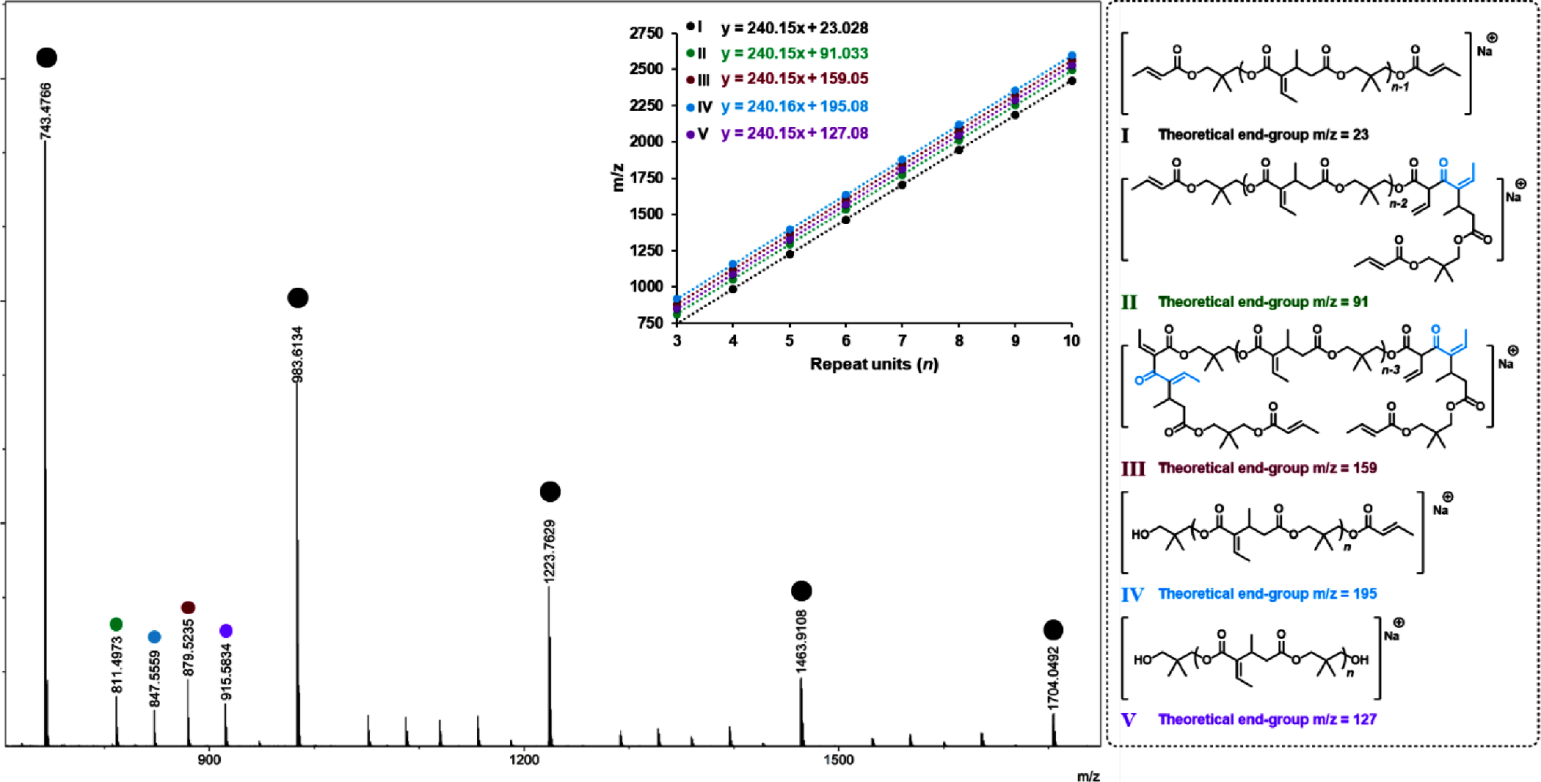
MALDI-TOF spectrum of poly­(DC-1) from Table 1, run 1, as well as corresponding end-group calculations and predicted structures **I–V**.

Importantly, Figure S22 shows the same MALDI data as Figure 3 but expanded to the higher-molecular-weight region (*m*/*z* = 4500–7000). In this range, the normal structure **I**—whether linear, cyclic, or both—is no longer the dominant species as it is in the lower-MW region (Figure 3) but instead appears at comparable abundance to the other Claisen condensation products **II**–**V**. This trend mirrors the GPC results, which reveal a substantial fraction of low-MW polymers regardless of concentration (Figure S23). The predominance of species **I** at low MWs followed by its decline at higher MWs suggests that the cyclization pathway is highly efficient at the early stages of growth but becomes less favored as chain length increases.

Given that the Claisen condensation accounts for a large fraction of the products, it is noteworthy that the alkoxide leaving group generated in this process is structurally similar to that of KO*^t^* Bu. Accordingly, it should retain sufficient basicity to deprotonate crotonate groups, thereby sustaining the concentration of catalytic enolate intermediate **6**. Nucleophilic substitution at the carbonyl by this primary alkoxide is inconsequential, as it simply regenerates the same alkoxide species. Thus, while the Claisen condensation pathway is unlikely to significantly affect *M*_n_, it may exert a modest influence on *M*_w_ and *Đ*. However, the increased acidity of the 1,3-dicarbonyl moiety (Figure 3, structure **III**) can lead to irreversible deprotonation, representing a plausible termination pathway by sequestering catalytic species. This would explain why 5–10 mol % of KO*^t^* Bu is necessary to reach high conversions while 1 mol % is insufficient (Tables 1 and 2).

Additionally, MALDI-TOF analysis was performed on poly­(DC-1) prepared with a high concentration of the end-capping agent methyl crotonate (MC) ([MC]_0_ = 16 mol % relative to [DC-1]_0_; Figures S18 and S19). Under these conditions, the main peak series corresponding to uncapped polymer (structure **I**) would be expected at low intensity due to the high likelihood of chain-end-capping by MC. Instead, peaks for structure **I** were observed at a relatively high intensity. Notably, a strong signal for polymer **I** at *m*/*z* 1223.74 (*n* = 5) is the most intense peak in the spectrum, despite the excess capping agent, suggesting that some chains escape end-capping by forming cyclic structures. As *n* increases, the intensity of this series (**I**) drops sharply, consistent with a higher probability of end-capping at greater DP and lower activity of cyclization at higher DP. If macrocyclization were absent, these low-*n* peaks would be expected to appear as capped species containing MC, rather than as abundant uncapped chains. While not conclusive, these results provide indirect evidence for macrocyclization, as intramolecular cyclization could occur before the chain end encounters the MC, thereby preventing capping.

Similarly, a MALDI-TOF spectrum was obtained for poly­(DC-2) from run 9 (Figure S17), which yielded comparable results. A dominant set of peaks corresponding to structure **A** (analogous to structure **I**) was observed with high intensity. Minor sets of peaks corresponding to Claisen condensation product **B** (analogous to **II**) and alcohol-terminated leaving group products **C** and **D** (analogous to **IV** and **V**) were also observed at lower intensity. Notably, no peaks consistent with elimination of the crotonate group from the bridging alkane were detected (theoretical end-group *m*/*z* = 163), suggesting that KO*^t^* Bu-catalyzed elimination is not operative under the conditions employed. This is consistent with previous findings by Waymouth,^68^ where the dimerization of *tert*-butyl crotonate and isopropyl crotonate proceeded efficiently with no evidence of elimination byproducts.

We reanalyzed the polymer from run 1 by MALDI-TOF after four months of ambient storage. Although the sample was quenched with benzoic acid, it was otherwise dried under a vacuum and stored in a 20 mL glass vial with a plastic cap. The MALDI spectrum (Figure S21) showed increased intensity of peaks corresponding to hydrolyzed structures, which were previously minor or absent in the freshly prepared sample. This observed degradation supports the potential of these polymers to undergo hydrolysis under environmental conditions. Furthermore, GPC analysis after four months of ambient storage reveals a decrease in *M*_n_ from 9.12 to 5.24 kg/mol and a reduction in dispersity from *Đ* = 2.07 to *Đ* = 1.79. These observations are consistent with chain scission over time and suggest promise for natural degradation in soil or water environments.

To obtain more direct evidence for macrocyclization, we employed transmission electron microscopy (TEM), a well-established technique for visualizing cyclic polymers.^71–75^ To enhance the contrast and apparent size of the polymer chains, we modified poly­(DC-1) via thiol–ene click chemistry by reacting it with 1-octadecanethiol and photoinitiator 2,2-dimethoxy-2-phenylacetophenone (DMPA). Since each repeat unit of poly­(DC-1) contains an alkene, this reaction yielded a polymer bearing an octadecane thioether pendant group on each repeat unit (Scheme 6). Successful modification was confirmed by the disappearance of alkene signals in the ^1^H NMR spectrum (Figure 4A). The addition of long C18 alkyl chains also gives the polymer crystalline behavior and a melting transition observable by DSC at 57 °C (Figure 4B)

**Scheme 6:**
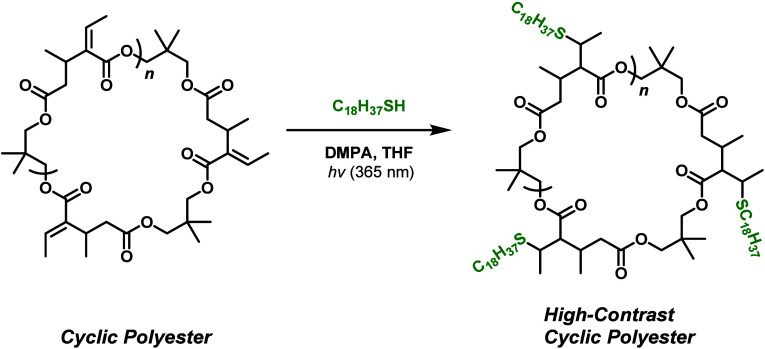
Postfunctionalization of Postulated Cyclic Poly­(DC-1) Using the Photo-Initiated Thiol–Ene Click Reaction

**Figure 4:**
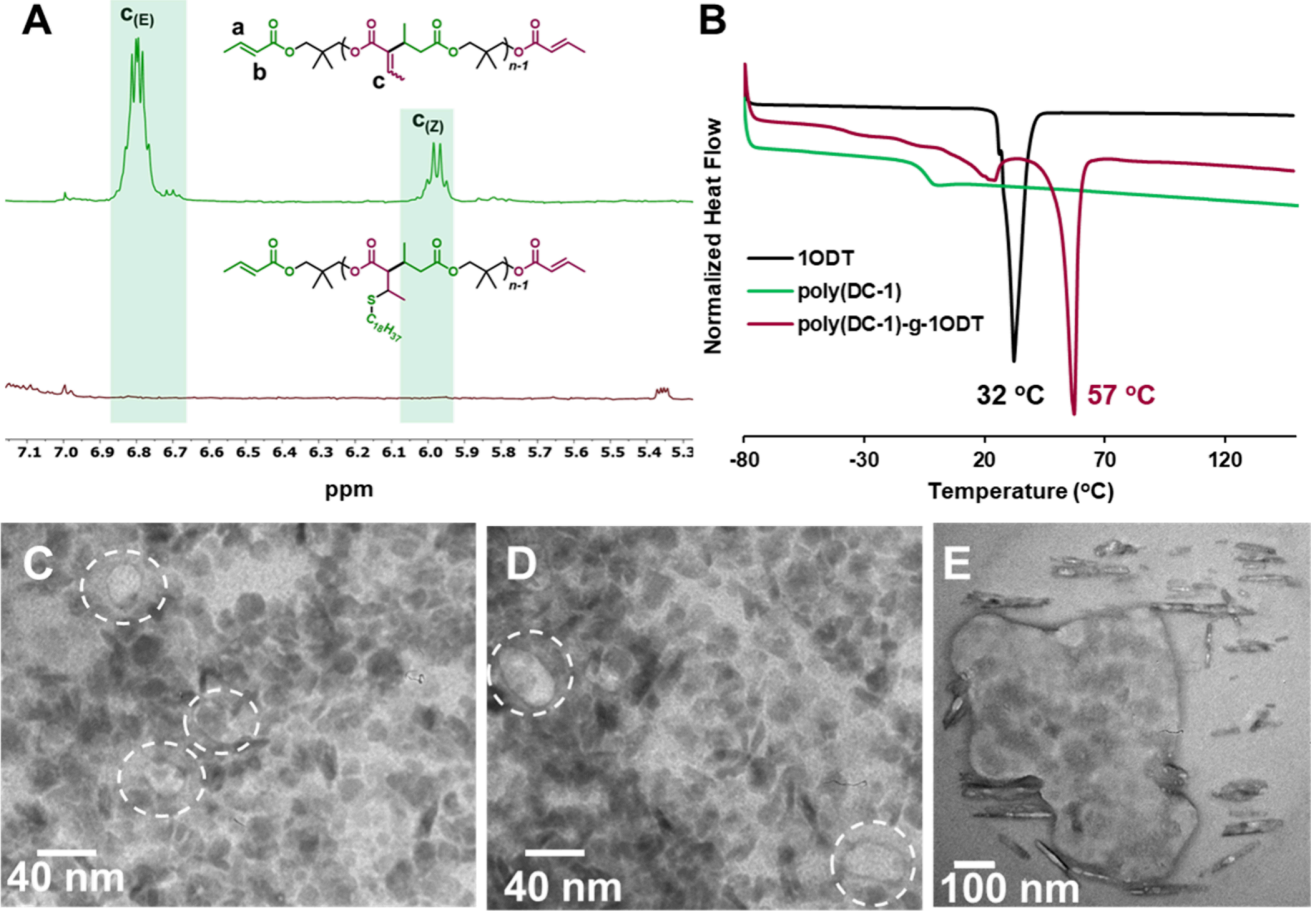
(Top row) Characterization of poly­(DC-1-*g*-1ODT)-**A**, including (A) stacked ^1^H NMR spectra before and after the thiol–ene reaction, expanded to the alkene region to show the disappearance of the *c* protons. (B) DSC traces of poly­(DC-1) before and after the thiol–ene reaction, with poly­(DC-1-*g*-1ODT)-**A** exhibiting a melting transition at *T*_m_ = 57 °C, distinct from both the parent poly­(DC-1) and the 1ODT control (*T*_m_ = 32 °C). (Bottom row) TEM images of poly­(DC-1-*g*-1ODT), including (C–D) images from poly­(DC-1-*g*-1ODT)-**A**, which is hypothesized to be enriched in a cyclic polymer. Emphasis is placed on circular structures approximately 40 nm in diameter. (E) Image from poly­(DC-1-*g*-1ODT)-**B**, hypothesized to be enriched in linear polymer, highlighting elongated crystalline domains approximately 100–200 nm in length.

For TEM analysis, we selected the poly­(DC-1) synthesized using normal 1 M THF conditions, 10 mol % KO*^t^* Bu akin to run 1 (*M*_n_ = 8.45 kg/mol before postfunctionalization) to make poly­(DC-1-*g*-1ODT)-**A**. As a control, we used a polymer which contains 4 mol % MC chain-end-capping agent and is therefore expected to favor a linear topology (poly­(DC-1-*g*-1ODT)-**B**, *M*_n_ = 6.47 kg/mol before postmodification). Both samples likely contain a mixture of linear and cyclic species but differ in the expected ratio. Previous studies using this TEM approach typically employ high-MW polymers to maximize imaging success.^74^ However, obtaining high-MW structures is difficult through the present polyaddition methodology due to (presumed) cyclization.

Nonetheless, Figure 4C,D (and Figures S29 and S30) shows TEM images of poly­(DC-1-*g*-1ODT)-**A**, which is predicted to be predominantly cyclic. Circular features approximately 40 nm in diameter are observed, consistent with the presence of cyclic polymer. While many polymers appear to crystallize into spherical domains, isolated ring-like structures are evident. In contrast, Figure 4E (and Figure S31) shows polymers from poly­(DC-1-*g*-1ODT)-**B**, which is expected to contain a higher proportion of linear chains due to the use of an end-capping agent. These samples exhibit larger crystalline domains, including elongated features suggestive of a linear polymer morphology.

Figure 4C–E shows our most objective and representative TEM images. Additional images are provided in Figures S32–S37 for readers to examine. These supplementary images, while suggestive of cyclic structures, were excluded from the main text due to various inconsistencies and uncertainties in order to maintain appropriate skepticism and avoid overpromoting a mechanism that remains speculative.

Lastly, thermal analysis of poly­(DC-1) and poly­(DC-2) was performed and is shown in Figure 5. Differential scanning calorimetry (Figure 5, left) revealed low *T*_g_’s for both polymers—*T*_g_ = – 5 °C for poly­(DC-1) and *T*_g_ = – 45 °C for poly­(DC-2)—likely due to the presence of alkene groups in the repeat units. These results indicate that the thermal properties of the material can be tuned by modifying the polymer backbone. Thermogravimetric analysis (TGA, Figure 5, right) showed comparable thermal stability for both polymers, with decomposition temperatures (*T*_d5_, defined as the temperature at 5 wt % mass loss) of 227 °C for poly­(DC-1) and 216 °C for poly­(DC-2).

**Figure 5:**
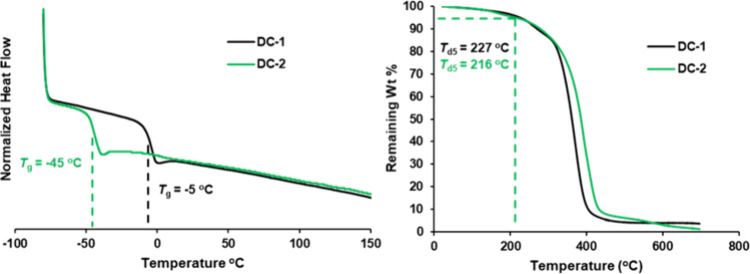
Thermal analysis for poly­(DC-1) and poly­(DC-2) polymers. (left) DSC profiles of both polymers and (right) TGA profiles of both polymers.

## Conclusions

We have demonstrated the successful synthesis and polymerization of DC-based monomers via a simple, yet effective, polyaddition strategy. The proposed mechanism involves deprotonation at the γ-position of the crotonate followed by Michael-type chain addition and chain transfer, resulting in linear polyesters under ambient conditions using inexpensive KO*^t^* Bu as catalyst. This method is both atom-economic and operationally straightforward, rapidly achieving high conversions. The resulting polyester platform is tunable through the variation of the diol-derived bridge, enabling control over structural and thermomechanical properties. Moreover, the system is fully biorenewable and degradable.

Additionally, evidence for a highly efficient macrocyclization pathway was observed, albeit indirectly. While still highly speculative, this mechanism is supported by multiple lines of evidence, including kinetic analysis, MALDI-TOF MS, and TEM imaging. This presumed cyclization process currently limits achievable *M*_n_ values. Future work will focus on developing more sophisticated catalytic systems to enable controlled, solvent-free polymerizations. This will not only enhance the sustainability of the process by eliminating solvent use but may also allow access to high molecular weight polymers.

## Supplementary Material



## Notes

Detailed experimental conditions, materials and methods as well as supplemental NMR and MS spectra for all monomers and polymers and additional SEM images of polymers (PDF)
