# Supplementary material for: Sustainable Polyaddition Path to Polyesters via Catalytic Homocoupling of Renewable Dicrotonate Monomers
Source: Macromolecules. 2025 Nov 30;58(23):12476–88. doi: 10.1021/acs.macromol.5c02308 (PMC12874653; doi:10.1021/acs.macromol.5c02308)
Supplement: Supplementary file 1 [file ma5c02308_si_001.pdf]

Supporting Information

A Sustainable Polyaddition Path to Polyesters via Catalytic Homocoupling of Renewable Di-Crotonate Monomers

Asif Shabbir, Mahsa Saeidi‡, Braden D. Pickle, Nicholas J. Tabbah, Michael L. McGraw*

Department of Chemistry and Biochemistry, University of Arkansas, Fayetteville, Arkansas, 72701, United States

‡Graduate School and International Education, Department of Materials Science & Engineering.

Table of Contents

A Sustainable Polyaddition Path to Cyclic and Linear Polyesters via Catalytic Homocoupling of DCs 1

Materials and Experimental Procedure 4

Materials...4

Synthesis of DC-1 4

Synthesis of DC-2 4

General Method of Polymerization 5

Analytical Methods...5

NMR Spectroscopy 5

Time of Flight Mass Spectrometry (TOF-MS)...5

Matrix Assisted Laser Desorption Ionization Time of Flight Mass Spectrometry (MALDI-TOF) 6

Gel Permeation Chromatography (GPC)...6

Thermal Analysis...6

Characterization 7

Figure S1: ^{1}H NMR ($CDCl_3$, RT) of DC-1...7

Figure S2: HH COSY spectrum of DC-1 in $CDCl_3$...8

Figure S3: ^{13}C NMR of DC-1 in $CDCL_3$...9

Figure S4: H NMR ($CDCl_3$, RT) of DC-2...10

Figure S5: HH COSY spectrum of DC-2 in $CDCl_3$...11

Figure S6: ^{13}C NMR of DC-2 in $CDCL_3$...12

Figure S7: ^{1}H NMR ($CDCl_3$, RT) of poly(DC-1) 13

Figure S8: HH COSY spectrum of poly(DC-1) 14

Figure S9: ^{13}C NMR of poly(DC-1) in $CDCL_3$..15

Figure S10: ^{1}H NMR ($CDCl_3$, RT) of poly (DC-2) 16

Figure S11: HH COSY spectrum of poly(DC-2) 17

Figure S12: ^{13}C NMR of poly(DC-2) in $CDCL_3$..18

Figure S13: LC-MS Spectrum of DC-1..18

Figure S14: LC-MS Spectrum of DC-2..19

Figure S15: MALDI-TOF-MS spectrum of poly(DC-2) 19

Figure S16: Different structures determined from MALDI-TOF-MS spectrum of Poly(DC-1) with high concentration of the end-capping agent MC ($[MC]_0$ % = 16 (See figure 17)...20

Figure S17: MALDI-TOF-MS spectrum of poly(DC-1) with high concentration of the end-capping agent MC ($[MC]_0$ % = 16 20

Figure S18: Different structures determined from MALDI-TOF-MS spectrum of poly(DC-1) after four months of ambient storage (see Figure S19)……………………………………………………....................................21

Figure S19: MALDI-TOF-MS spectrum of Poly(DC-1) after four months of ambient storage……………..……22

Figure S20: Elution time of DC-1 from Flash chromatography with 75:25 hexane and ethyl acetate respectively………..22

Figure S21: Elution time of DC-2 from Flash chromatography with 75:25 hexane and ethyl acetate respectively…….….23

Preparation of 1-octadecanethiol-grafted DC-1 and DC-2 polymers…………………………………………………..23

Figure S22: ^{1}H NMR ($CDCl_3$, RT) of poly(DC-1) before and after thiol-ene click reaction ………….…………..24

Figure S23: dRI vs concentration plot used to measure exact dn/dc value for poly(DC-1), with calculated dn/dc = 0.0729 mL/g……….24

Figure S24: dRI vs concentration plot used to measure exact dn/dc value for poly(DC-2), with calculated dn/dc = 0.0647 mL/g………………………………………………………………………..…………………………………………..….25

Figure S25: Transmission electron microscopy (TEM) image of 1-octadecanethiol-grafted poly(DC-1) ...26

Figure S26: TEM image of 1-octadecanethiol-grafted poly(DC-1) (Table 4, run 33), labeled with guiding circles……...27

Figure S27: TEM image of 1-octadecanethiol-grafted poly(DC-1) (Table 4, run 36)…………………………………28

Materials and Experimental Procedure

Materials

Crotonic acid, potassium tert-butoxide (KO^{t}Bu), and potassium bis(trimethylsilyl)amide (KHMDS) were purchased from Thermo Scientific as 1 M stock solutions in THF and used as received. 2,2-Dimethylpropanol and 1,4-butanediol were obtained from Sigma-Aldrich and used without further purification. 1-Octadecanethiol ($CH_3(CH_2)_{17}SH$) was purchased from Ambeed, Inc. and used as received. Tetrahydrofuran (THF), toluene, and sulfuric acid were purchased from J.T. Baker and used as received. THF for polymerizations was HPLC grade and unstabilized, purified by distillation over CaH_2. Monomers DC-1 and DC-2 were synthesized via condensation of crotonic acid with 2,2-dimethylpropanol and 1,4-butanediol, respectively. Purified DC-1 and DC-2 products were dried over calcium hydride (Thermo-Scientific 0-0.2 mm grain size), for several hours and filtered through 0.45 µm nylon syringe filters inside a N_2-filled glovebox.

Synthesis of DC-1

In a 1000 mL round-bottom flask, 40.0 g of 2,2-dimethylpropanol (0.3841 mol) and 82.7 g of crotonic acid (0.9601 mol, 2.5 equiv) were dissolved in toluene under stirring. After complete dissolution, 1.5 mL of sulfuric acid was added as a catalyst. A Dean–Stark apparatus was employed to continuously remove the water byproduct, and the reaction mixture was refluxed for 8 hours. A total of 13.9 mL of water was collected, indicating near-complete conversion. Upon completion, toluene was removed under reduced pressure using a rotary evaporator.

The crude product was dissolved in ethyl acetate and washed once with water and three times with saturated sodium bicarbonate solution. The organic layer was dried over anhydrous sodium sulfate and concentrated under reduced pressure to afford the crude product (88.65 g, 96% yield). Purification was achieved by either distillation or flash column chromatography (25% ethyl acetate in hexanes). The purified monomer was dried over calcium hydride and stored under inert atmosphere for subsequent polymerization. Product identity and purity were confirmed by ^{1}H NMR, ^{13}C NMR, and mass spectrometry.

Synthesis of DC-2

In a 1000 mL round-bottom flask equipped with a magnetic stir bar, 39.21 mL of 1,4-butanediol (0.4438 mol) and 95.34 g of crotonic acid (1.1074 mol, 2.5 equiv) were dissolved in toluene. After complete dissolution, 1.5 mL of sulfuric acid was added as a catalyst. The mixture was heated to reflux, and a Dean–Stark apparatus was used to continuously remove the water byproduct. The

reaction proceeded for 8 hours, during which 15.9 mL of water was collected, consistent with the theoretical yield for complete diester formation.

After cooling to room temperature, the mixture was concentrated under reduced pressure to remove toluene. The residue was dissolved in ethyl acetate and subjected to liquid–liquid extraction: once with deionized water to remove excess acid, followed by three washes with saturated aqueous sodium bicarbonate to neutralize remaining acidic species. The organic layer was dried over anhydrous sodium sulfate, filtered, and concentrated under reduced pressure to afford the crude product (88.34 g, 88% yield).

Purification was performed by flash column chromatography using a mobile phase of 25% ethyl acetate in 75% hexanes. The product identity and purity were confirmed by ^{1}H NMR, ^{13}C NMR, and mass spectrometry. The purified diester (DC-2) was dried under reduced pressure over calcium hydride and stored under inert atmosphere for subsequent polymerization.

General Method of Polymerization

Polymerizations were conducted at room temperature in a nitrogen-filled glovebox using 25 mL glass vials. Prior to use, the vials were vacuum-purged and flushed with nitrogen for 10 minutes to remove oxygen and moisture, both of which can inhibit polymerization. A series of reactions were performed using 10 mol% potassium tert-butoxide (KO^{t}Bu) as a catalyst with varying amounts of DC monomers. In select reactions, 5 mol% potassium bis(trimethylsilyl)amide (KHMDS) was employed as an alternative catalyst. The resulting polymers were characterized by ^{1}H and ^{13}C NMR spectroscopy, matrix-assisted laser desorption/ionization time-of-flight mass spectrometry (MALDI-TOF), gel permeation chromatography (GPC), and thermal analysis.

Analytical Methods

NMR Spectroscopy

The synthesis of DC-1 and DC-2, as well as the polymerization of the DCs, was monitored and characterized by ^{1}H NMR and ^{13}C NMR spectroscopy using a Bruker BioSpin AV III 400 MHz spectrometer. Chemical shifts were referenced to the residual solvent signal of $CDCl_3$ at 7.26 parts per million (ppm) and reported in ppm relative to tetramethylsilane ($SiMe_4$).

Time of Flight Mass Spectrometry (TOF-MS)

Mass spectra of DC-1 and DC-2 were collected using a Shimadzu LCMS-IT-TOF mass spectrometer equipped with a Shimadzu C18 column (3 µm, 50 × 4.6 mm). The mobile phases consisted of water with 0.1% formic acid (solvent A) and acetonitrile with 0.1% formic acid (solvent B). The gradient program began at 30% B, held for 1 minute, then increased linearly to 90% B over 11 minutes. This composition was held for 1 minute before re-equilibrating to 30% B over 2 minutes, resulting in a total run time of 15 minutes. Mass spectra were acquired in positive-ion mode.

Matrix Assisted Laser Desorption Ionization Time of Flight Mass Spectrometry (MALDI-TOF)

Low molecular weight polymer samples were analyzed by matrix-assisted laser desorption/ionization time-of-flight (MALDI-TOF) mass spectrometry using a Bruker Ultraflex II (Bruker Daltonics GmbH, Bremen, Germany) instrument operated in positive-ion reflection mode. The system was equipped with a Nd:YAG laser (355 nm) and operated at an accelerating voltage of 25 kV. Raw spectral data were processed using FlexAnalysis software (version 2.4, Bruker Daltonics), and mass spectra were visualized and plotted using mMass software.

Gel Permeation Chromatography (GPC)

Absolute number-average molecular weight (M_n), weight-average molecular weight (M_w), and dispersity ($Đ = M_w/M_n$) of the polymers were determined by gel permeation chromatography (GPC). Measurements were carried out using a Waters Arc HPLC system equipped with three mixed-bed columns of different pore sizes for improved resolution. Tetrahydrofuran (THF) was used as the eluent at a flow rate of 1.0 mL/min at room temperature. The system was equipped with a Waters 2489 UV/Vis detector, a Wyatt Technology Optilab differential refractive index (dRI) detector, and a Wyatt Technology DAWN 8 multi-angle light scattering (MALS) detector. The refractive index increments (dn/dc) used for molecular weight determination were 0.0729 mL/g for DC-1-based polymers and 0.0647 mL/g for DC-2-based polymers.

Thermal Analysis

Differential scanning calorimetry (DSC) was performed using a TA Instruments DSC Q2500 with TZero aluminum pans. Samples were subjected to three thermal cycles: heating from 25 °C to 200 °C (cycle 1), cooling to −80 °C (cycle 2), and reheating to 200 °C (cycle 3), all at a rate of 10 °C/min. Only the second heating cycle (cycle 3) is shown in Figure 5.

Thermogravimetric analysis (TGA) was conducted on a TA Instruments TGA Q50. Samples were heated from 25 °C to 700 °C at a rate of 10 °C/min under a nitrogen atmosphere.

Characterization

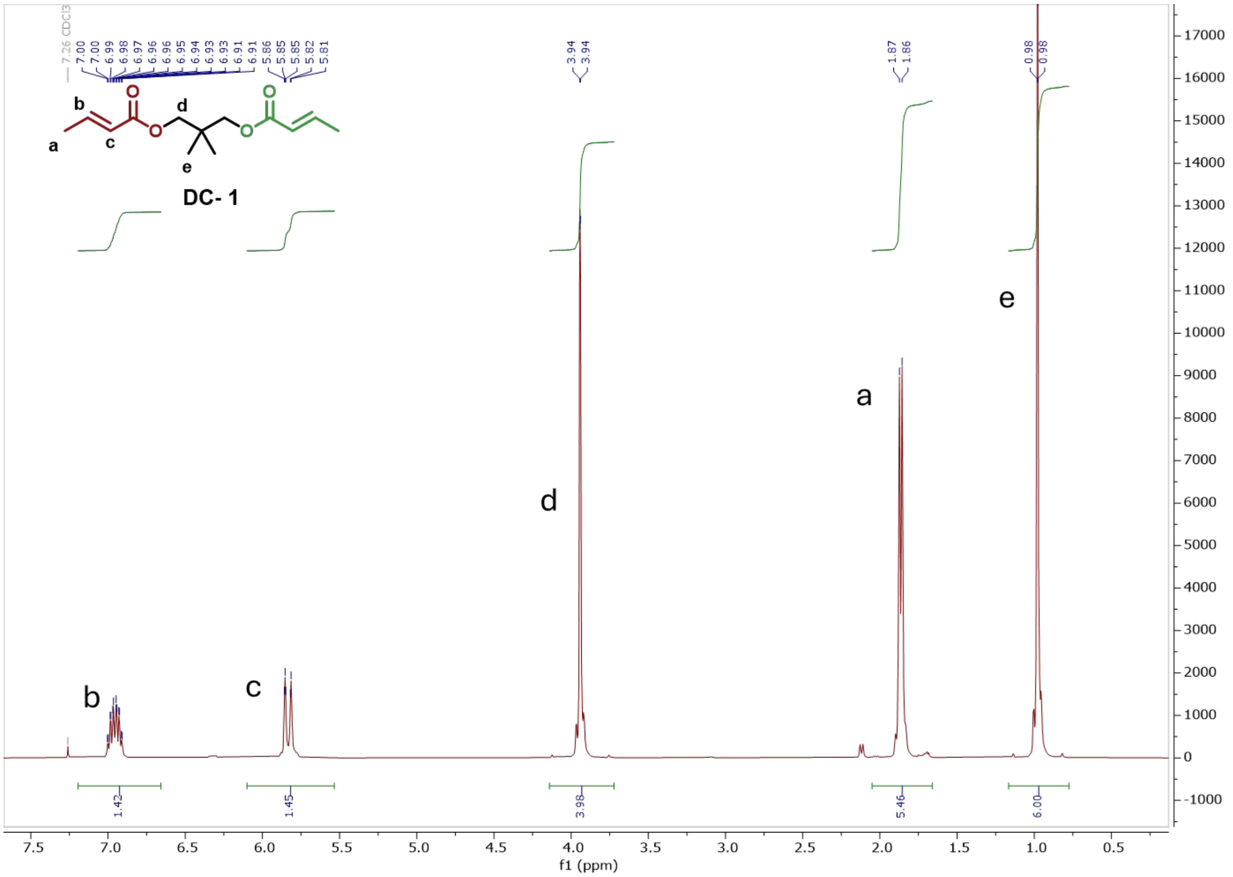

Figure S1. ^{1}H NMR ($CDCl_3$, RT) of DC-1.

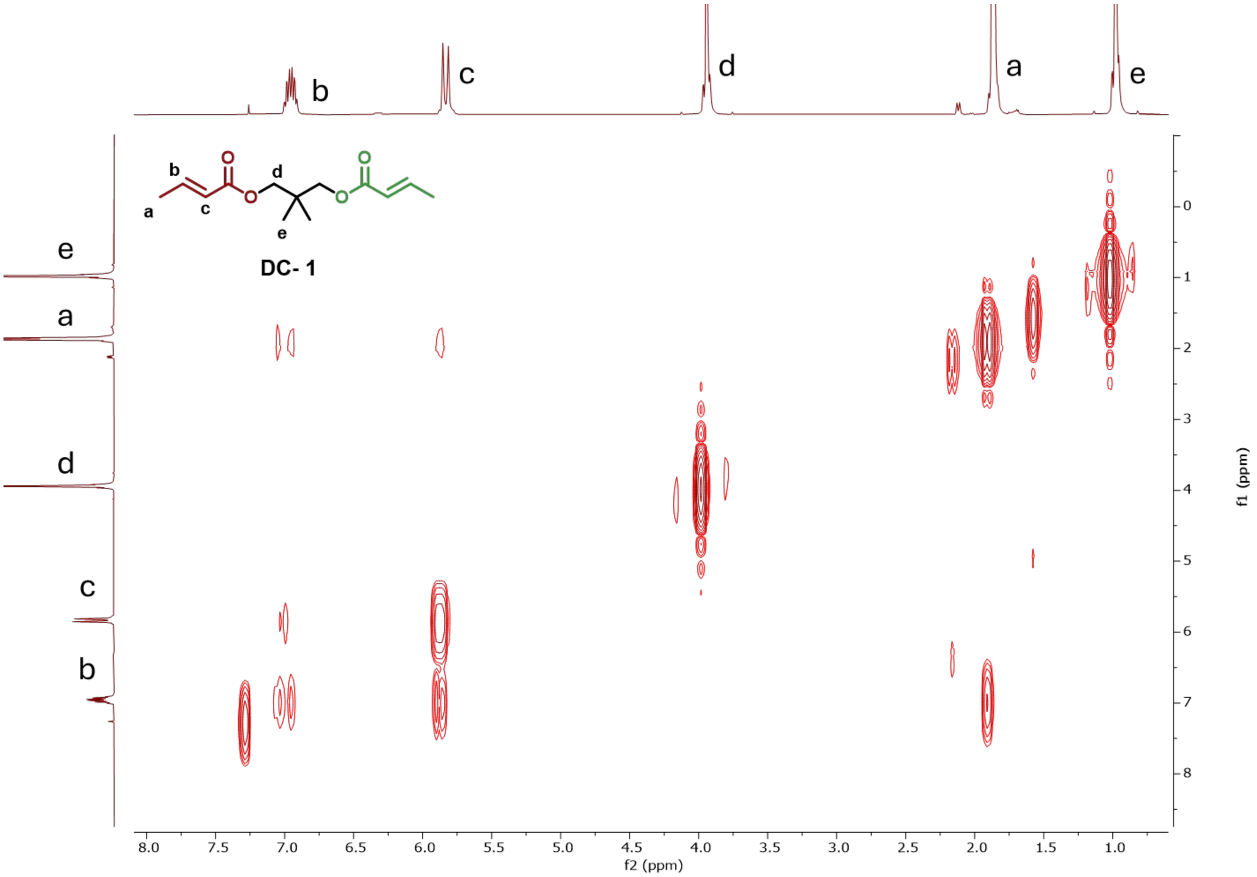

Figure S2. HH COSY ($CDCl_3$, RT) spectrum of DC-1.

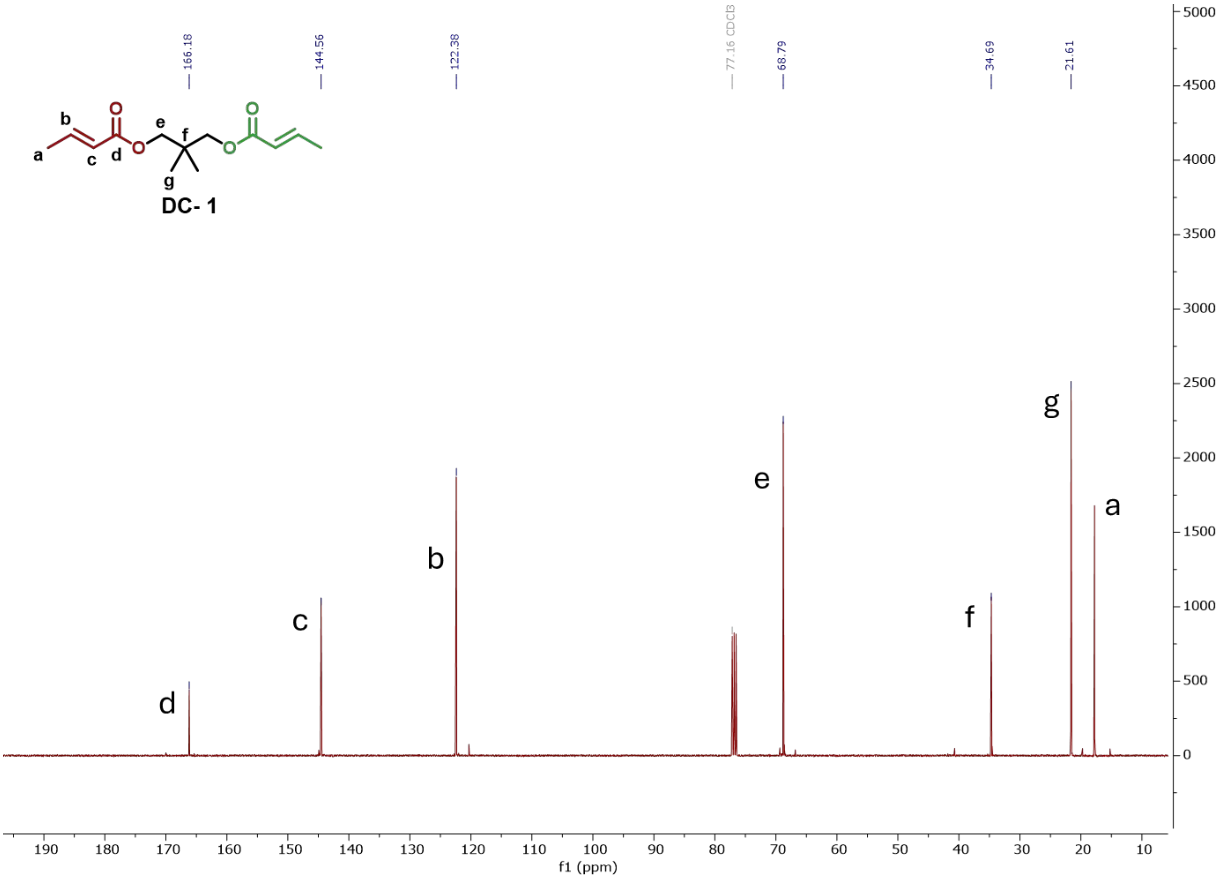

Figure S3. ^{13}C NMR ($CDCl_3$, RT) of DC-1.

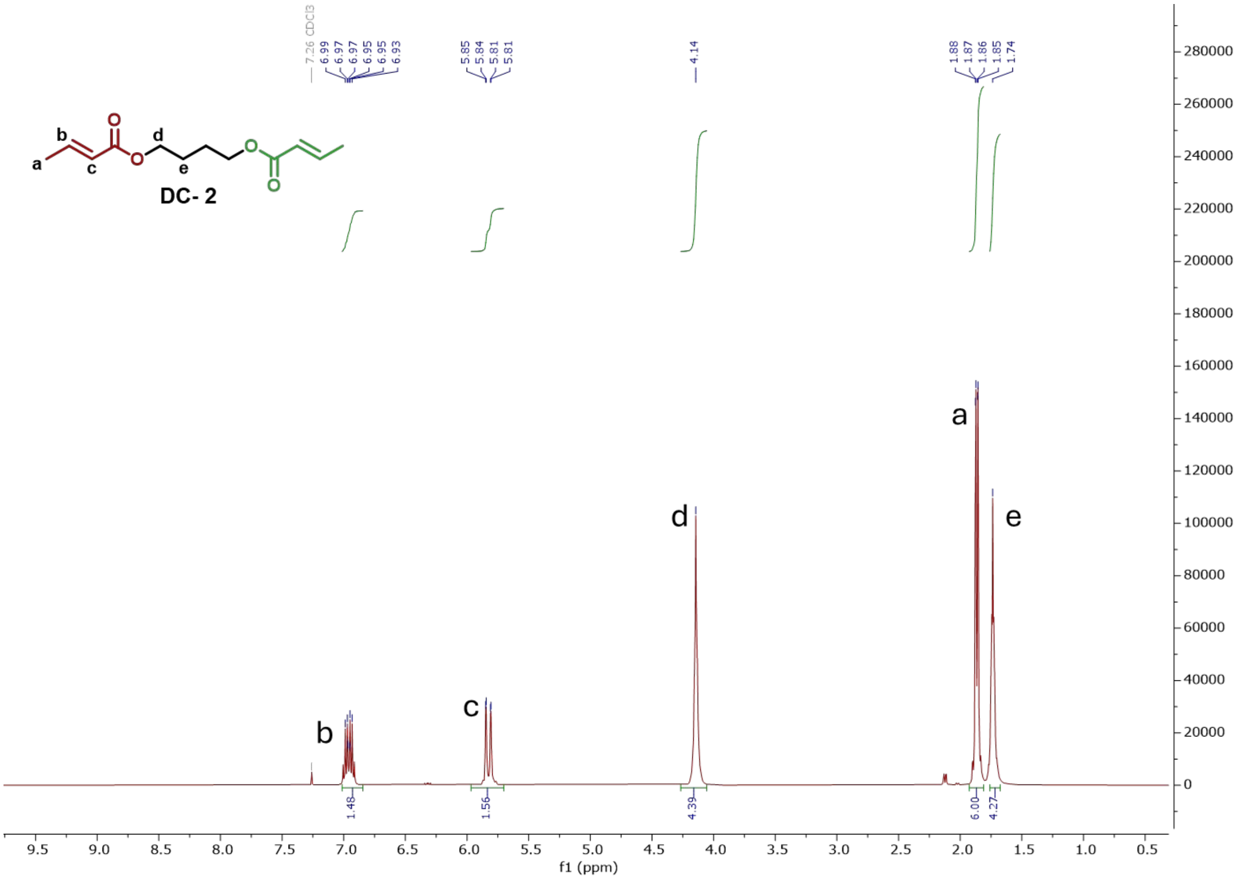

Figure S4. ^{1}H NMR ($CDCl_3$, RT) of DC-2.

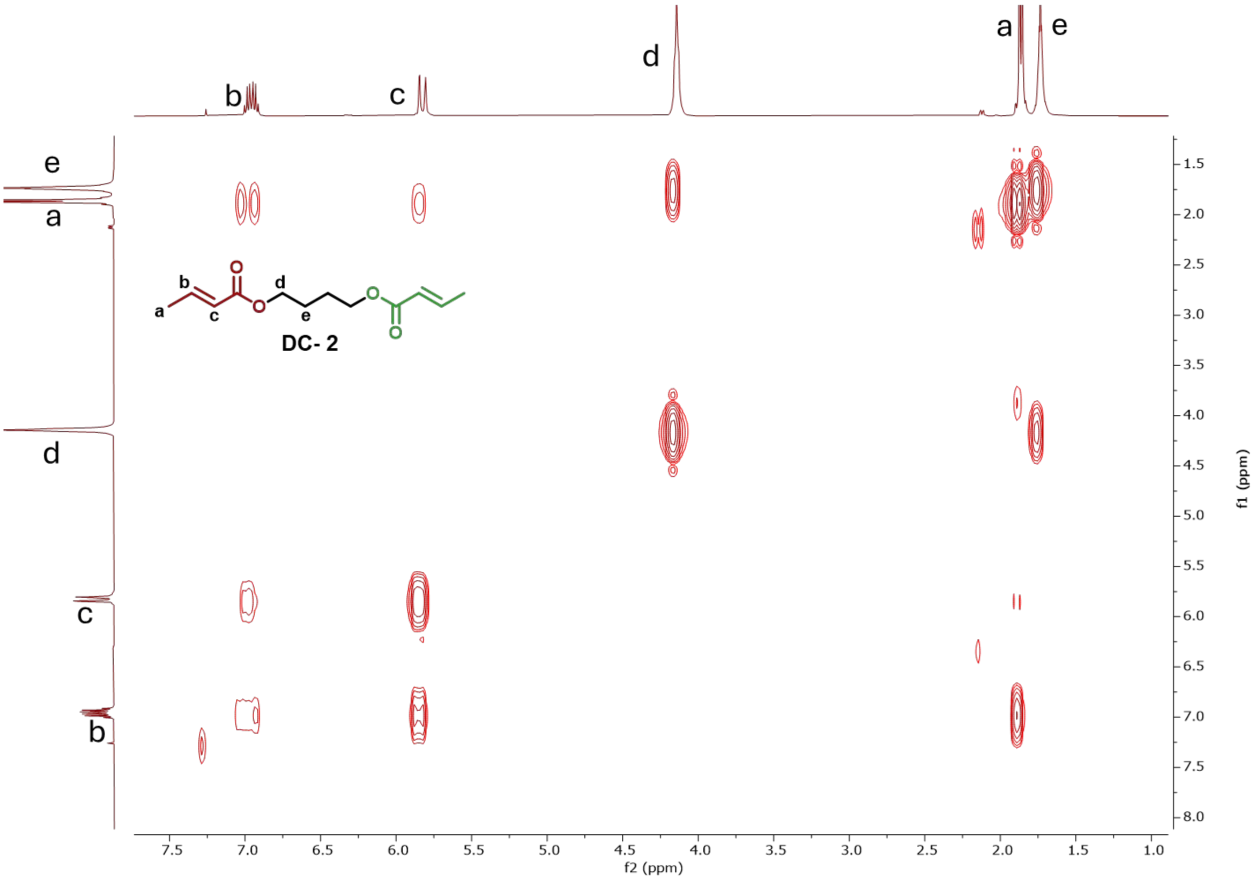

Figure S5. HH COSY ($CDCl_3$, RT) spectrum of DC-2.

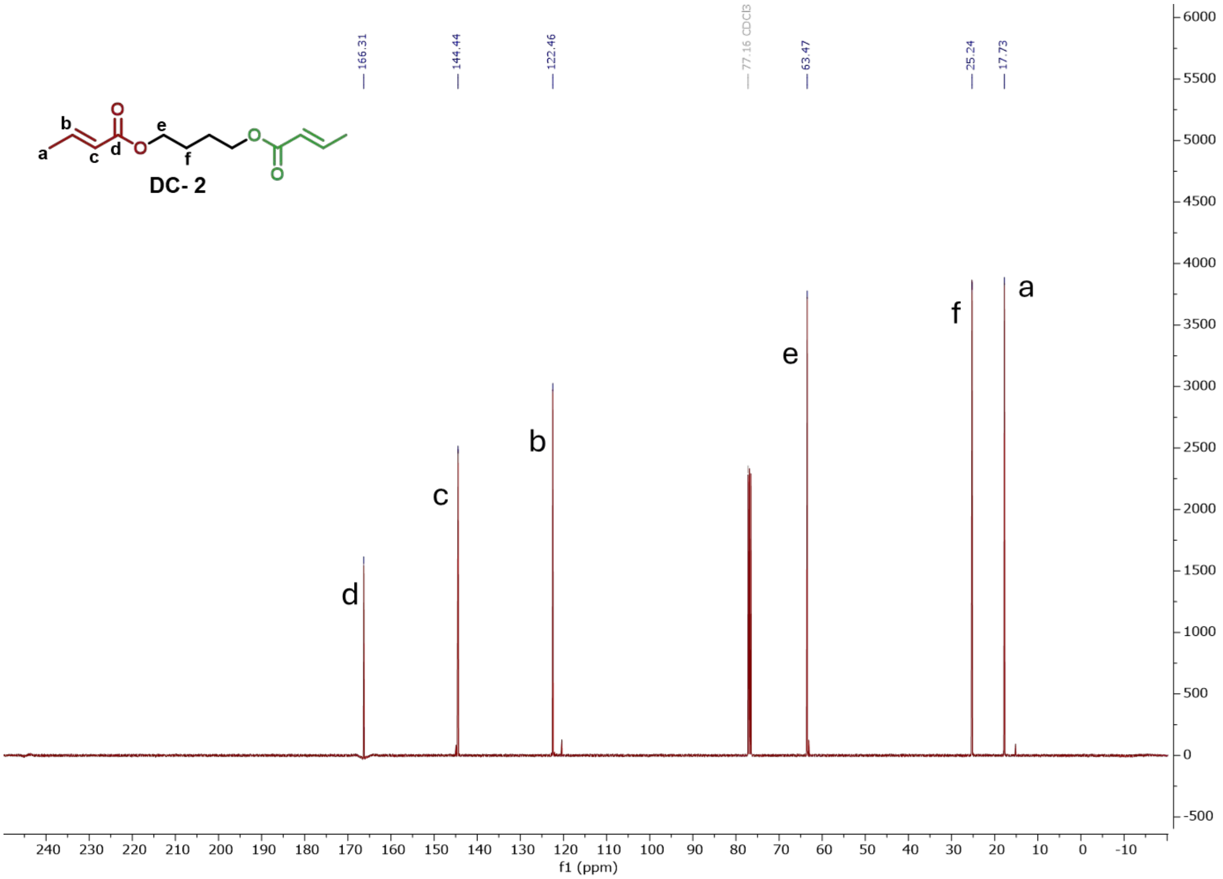

Figure S6. ^{13}C NMR ($CDCl_3$, RT) of DC-2.

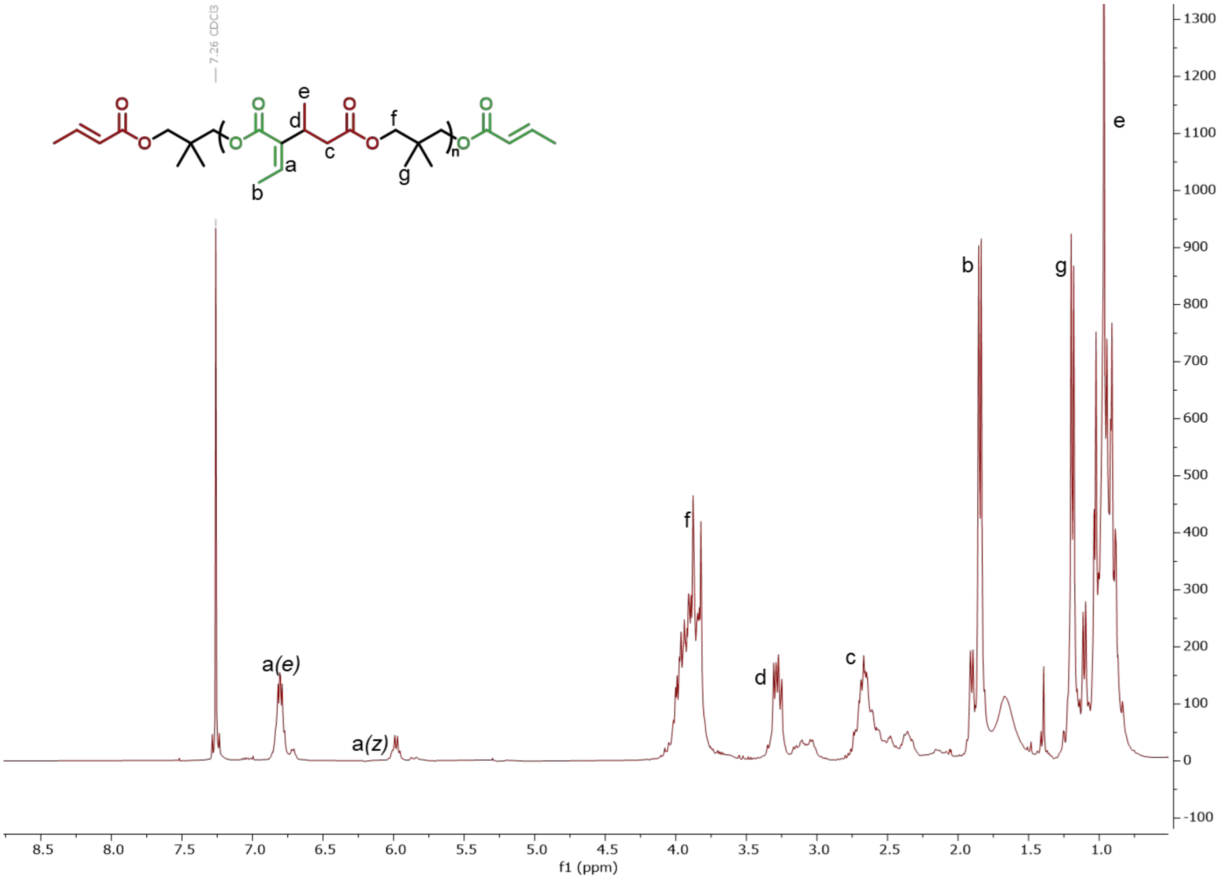

Figure S7. ^{1}H NMR ($CDCl_3$, RT) of poly(DC-1).

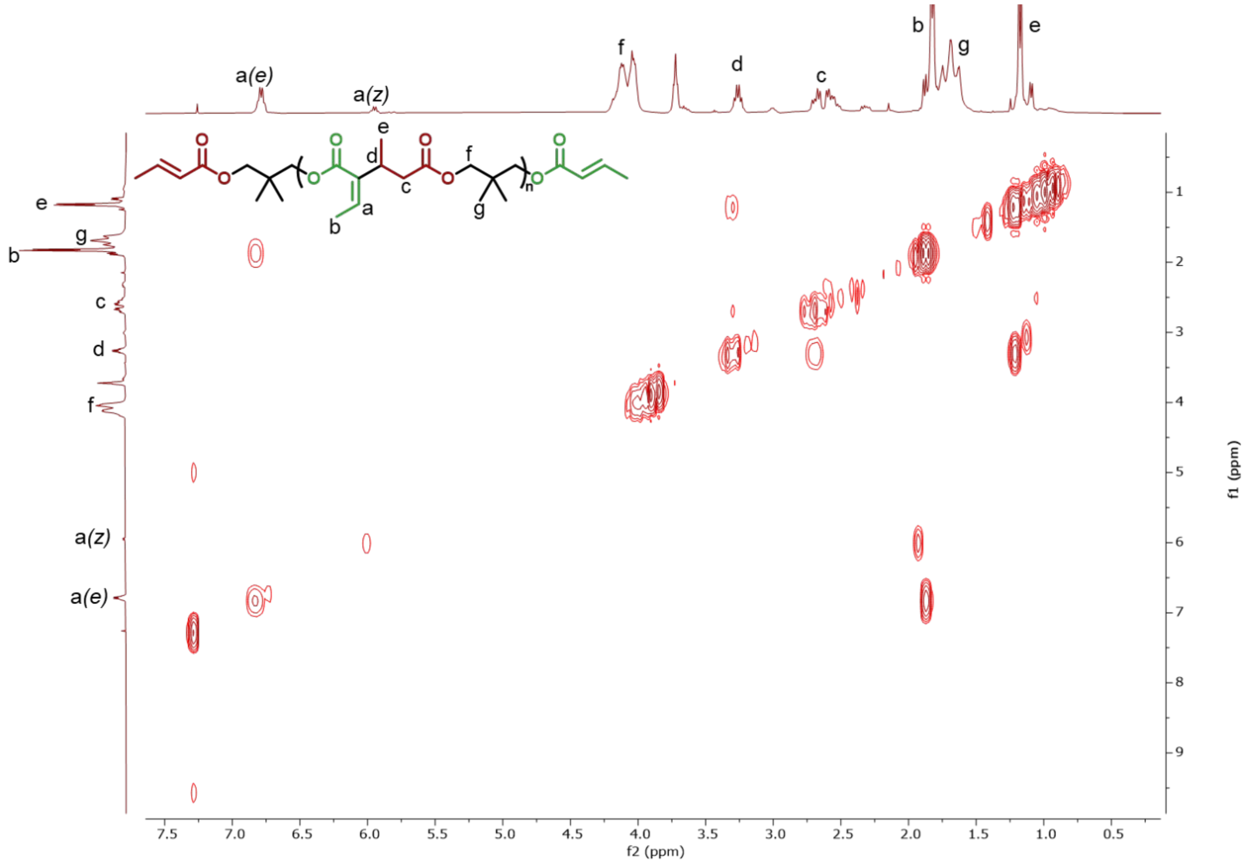

Figure S8. HH COSY ($CDCl_3$, RT) spectrum of poly(DC-1).

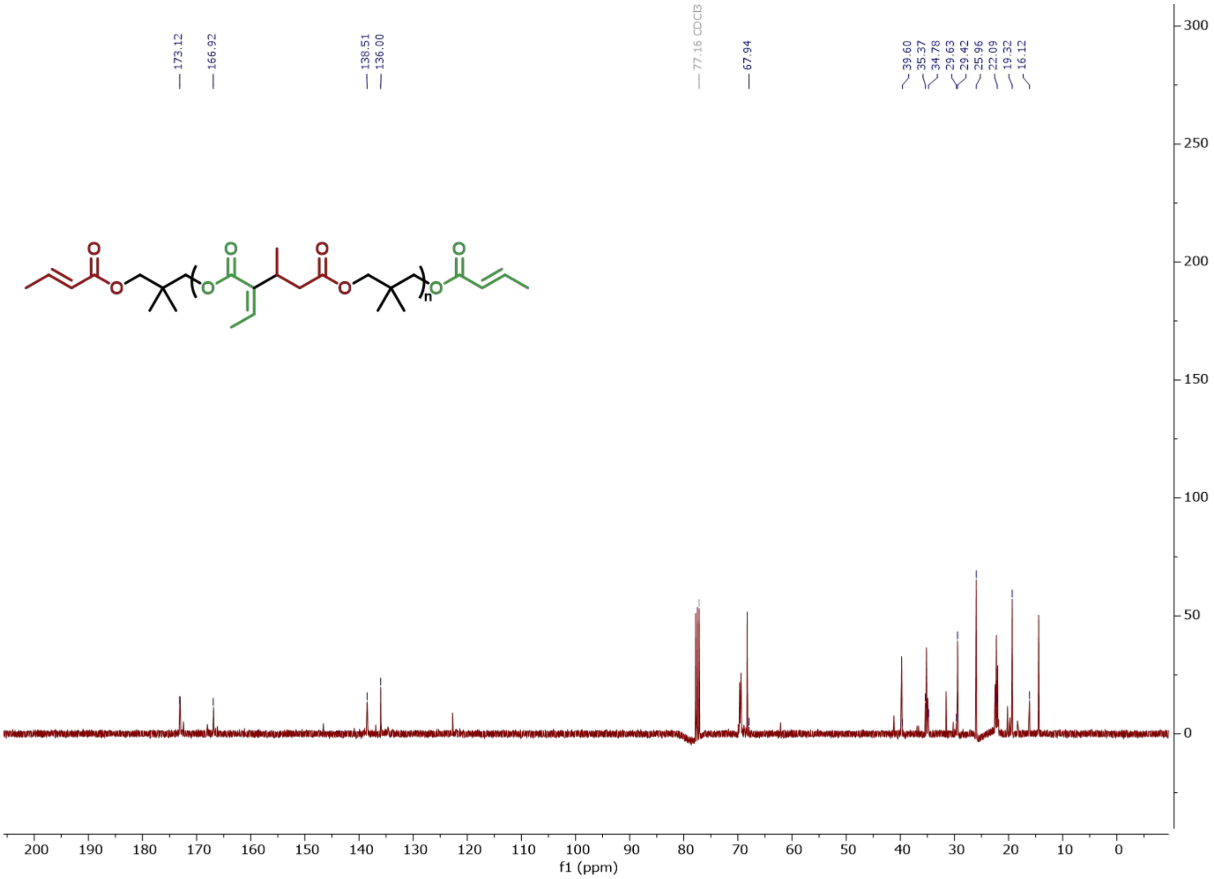

Figure S9. ^{13}C NMR ($CDCl_3$, RT) of poly(DC-1).

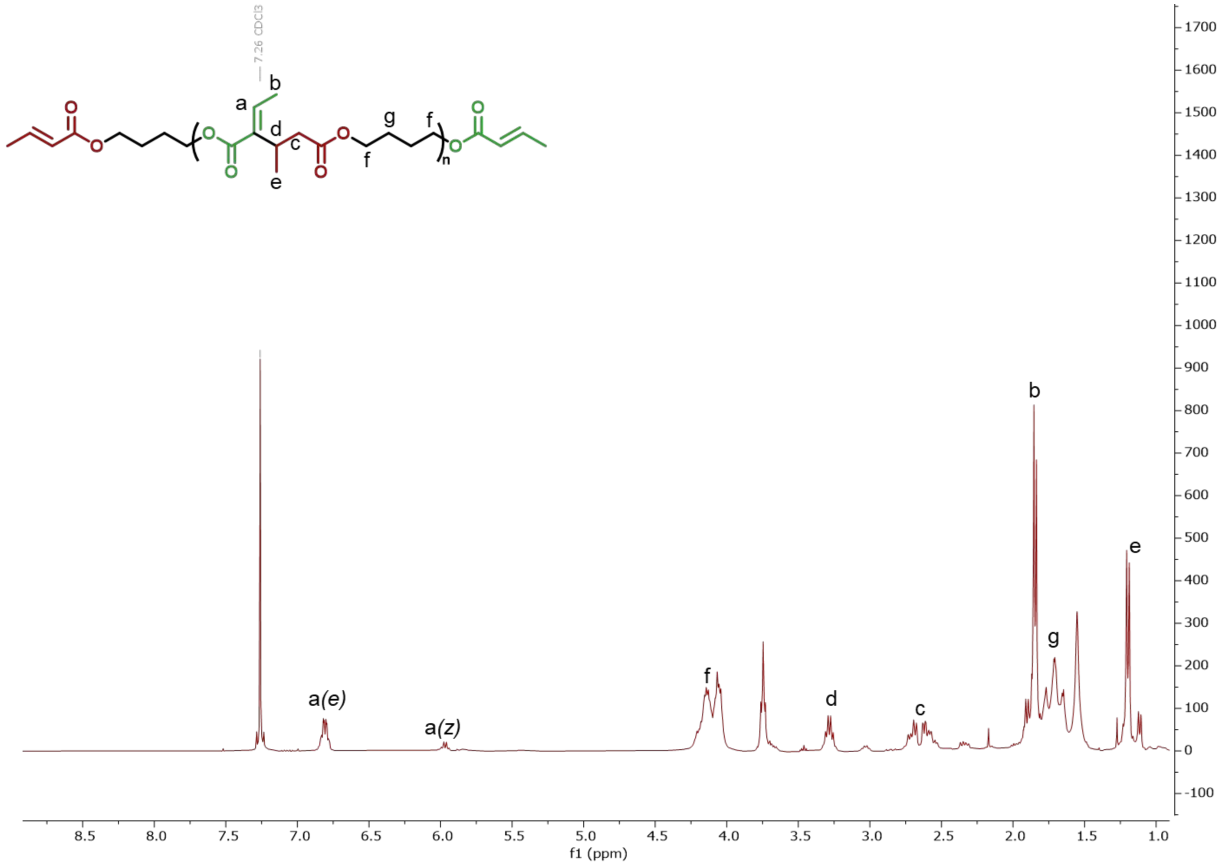

Figure S10. ^{1}H NMR ($CDCl_3$, RT) of poly(DC-2).

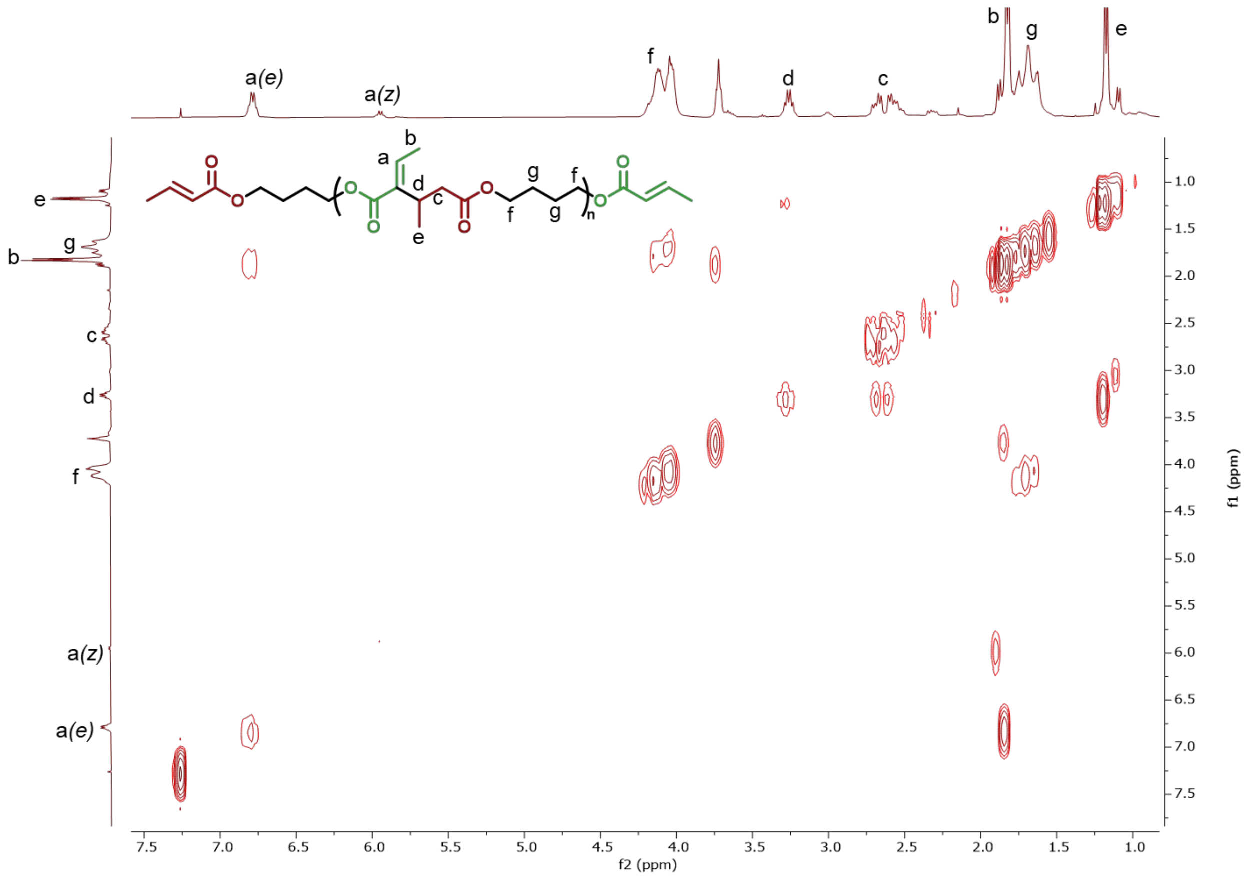

Figure S11. HH COSY ($CDCl_3$, RT) spectrum of poly(DC-2).

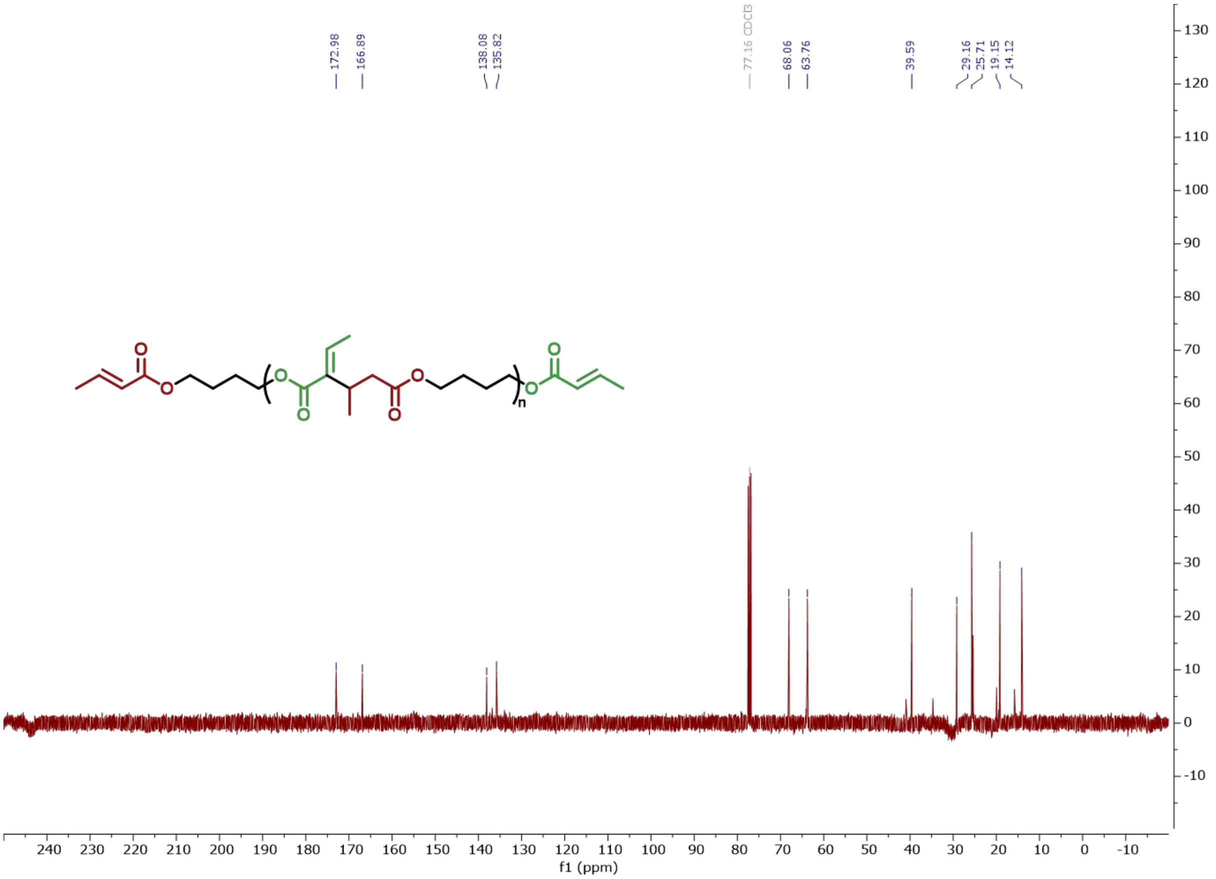

Figure S12. ^{13}C NMR ($CDCl_3$, RT) of poly(DC-2).

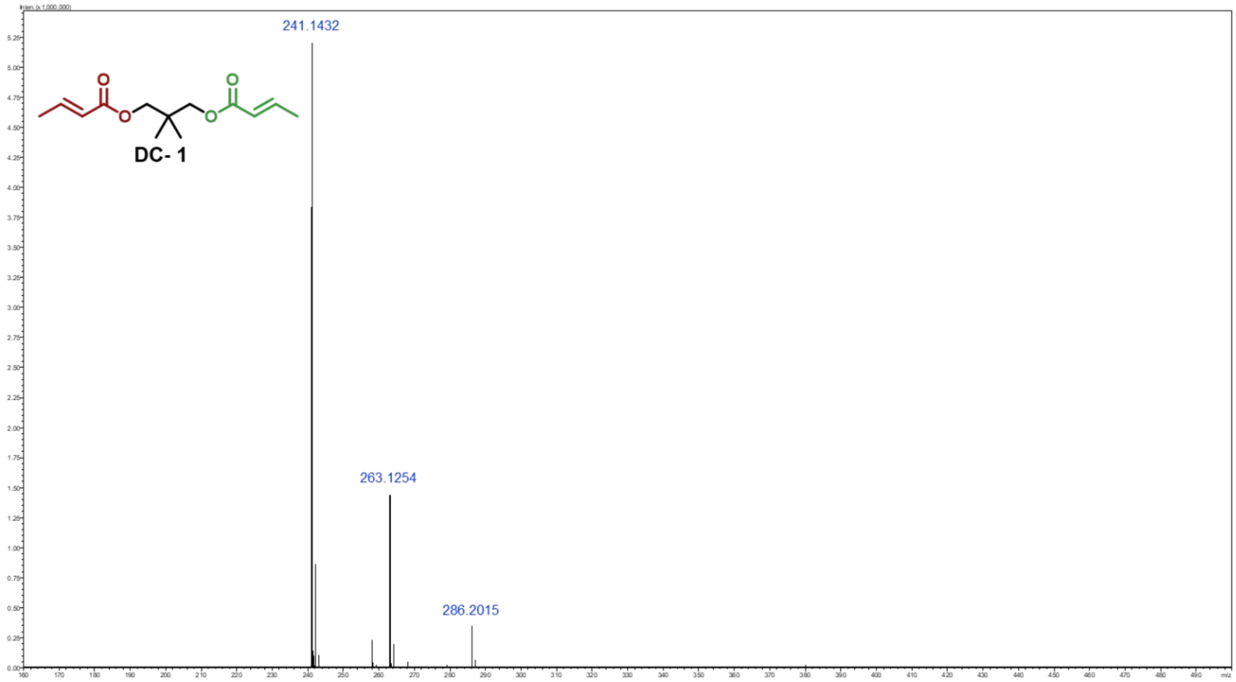

Figure S13. LC-MS Spectrum of DC-1.

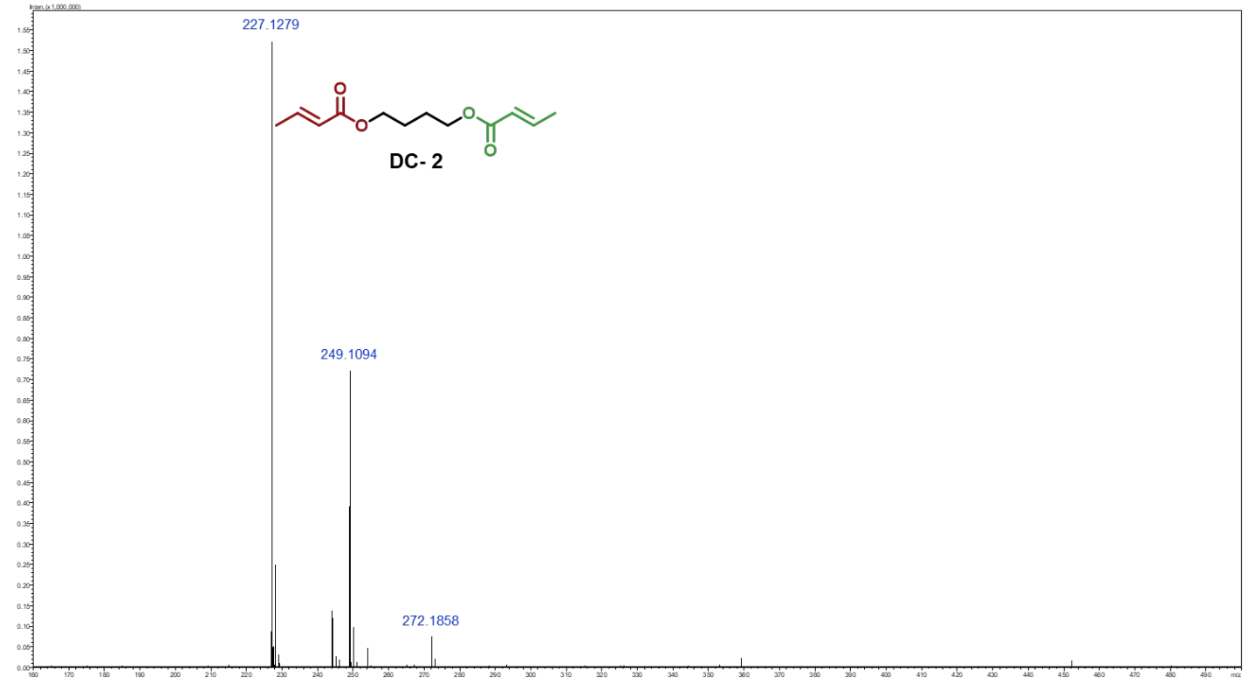

Figure S14. LC-MS Spectrum of DC-2.

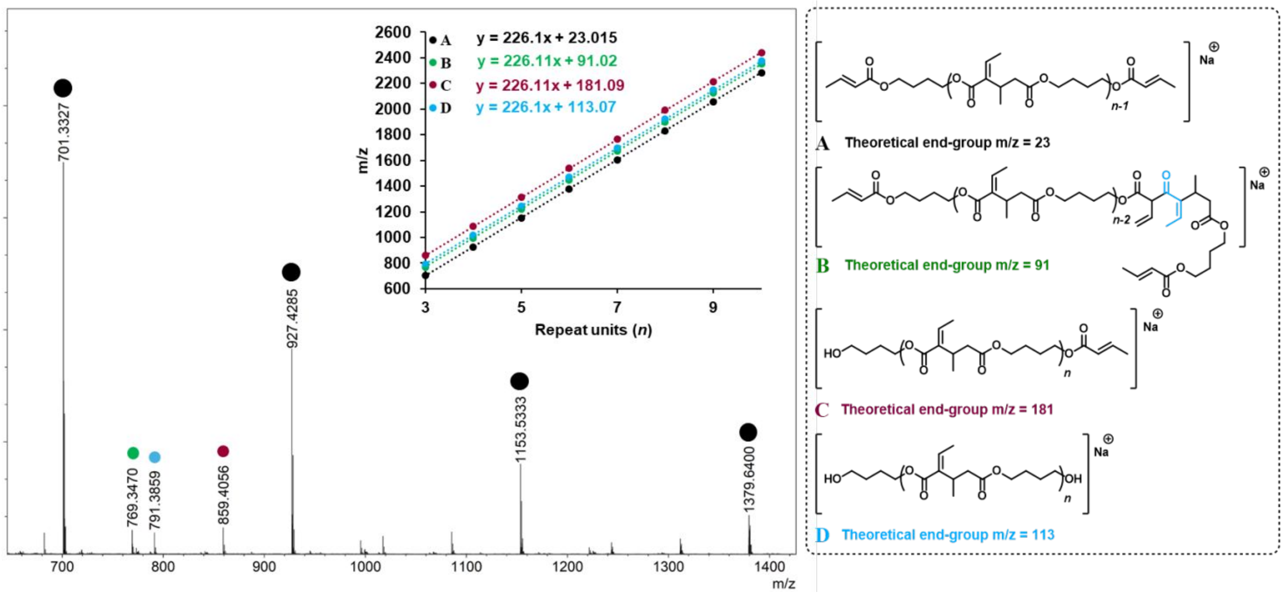

Figure S15. MALDI-TOF-MS spectrum of poly(DC-2), quenched after 5 minutes at more than 99 % conversion.

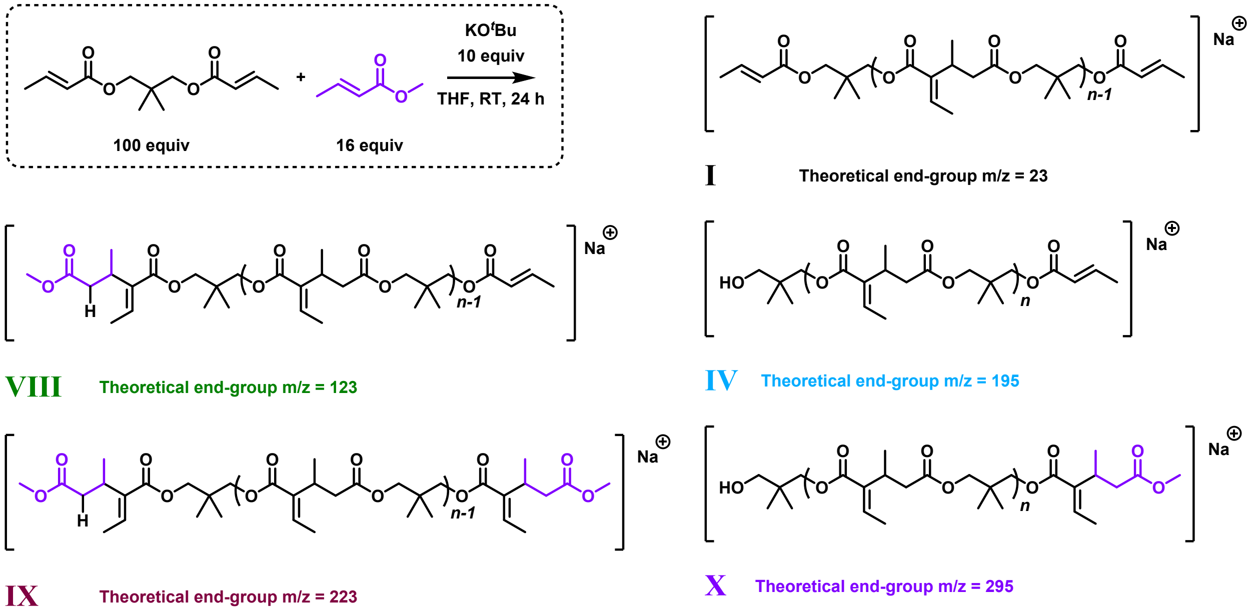

Figure S16. Different structures determined from MALDI-TOF-MS spectrum of poly(DC-1) with high concentration of the end-capping agent MC ($[MC]_0$ % = 16 (See Figure S17).

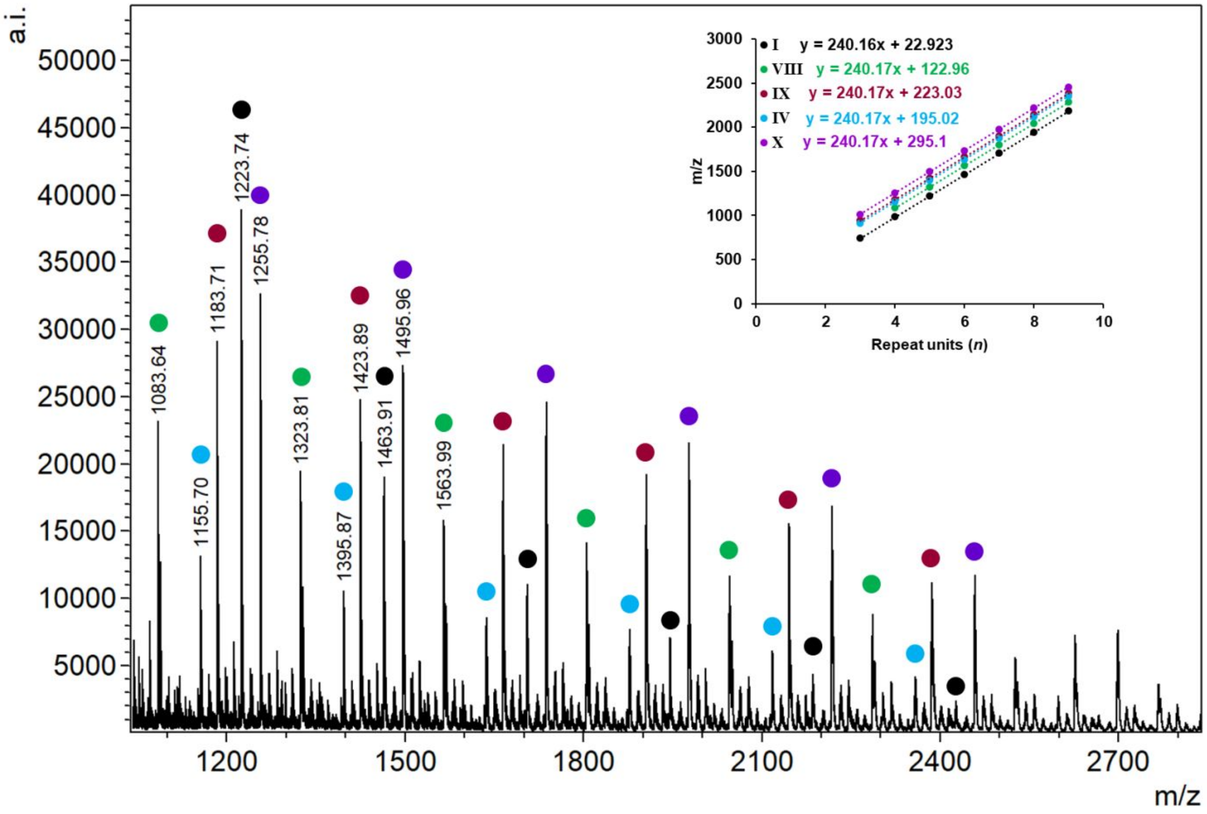

Figure S17. MALDI-TOF-MS spectrum of poly(DC-1) with high concentration of the end-capping agent MC ($[MC]_0$ % = 16).

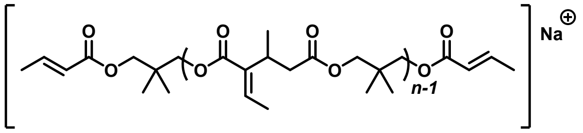

I Theoretical end-group m/z = 23

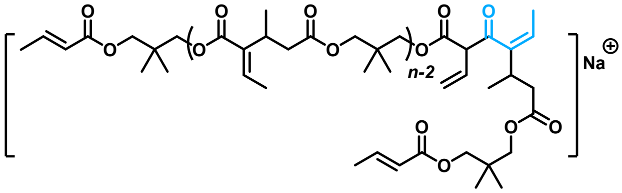

II Theoretical end-group m/z = 91

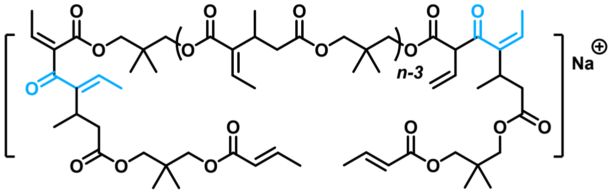

III Theoretical end-group m/z = 159

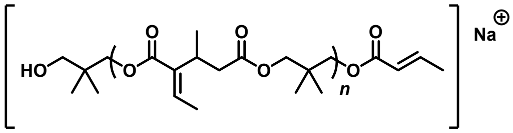

IV Theoretical end-group m/z = 195

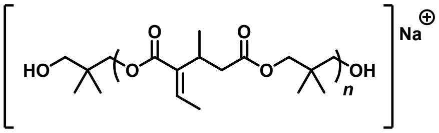

V Theoretical end-group m/z = 127

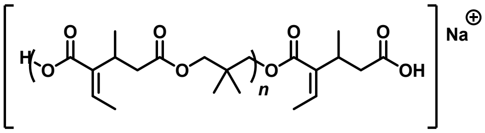

IV' Theoretical end-group m/z = 195

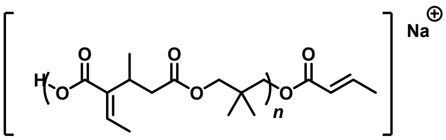

VI Theoretical end-group m/z = 109

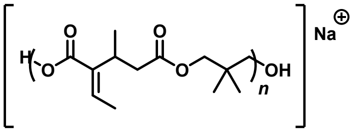

VII Theoretical end-group m/z = 41

Figure S18. Different structures determined from MALDI-TOF-MS spectrum of Poly(DC-1) after four months of ambient storage (see Figure S19).

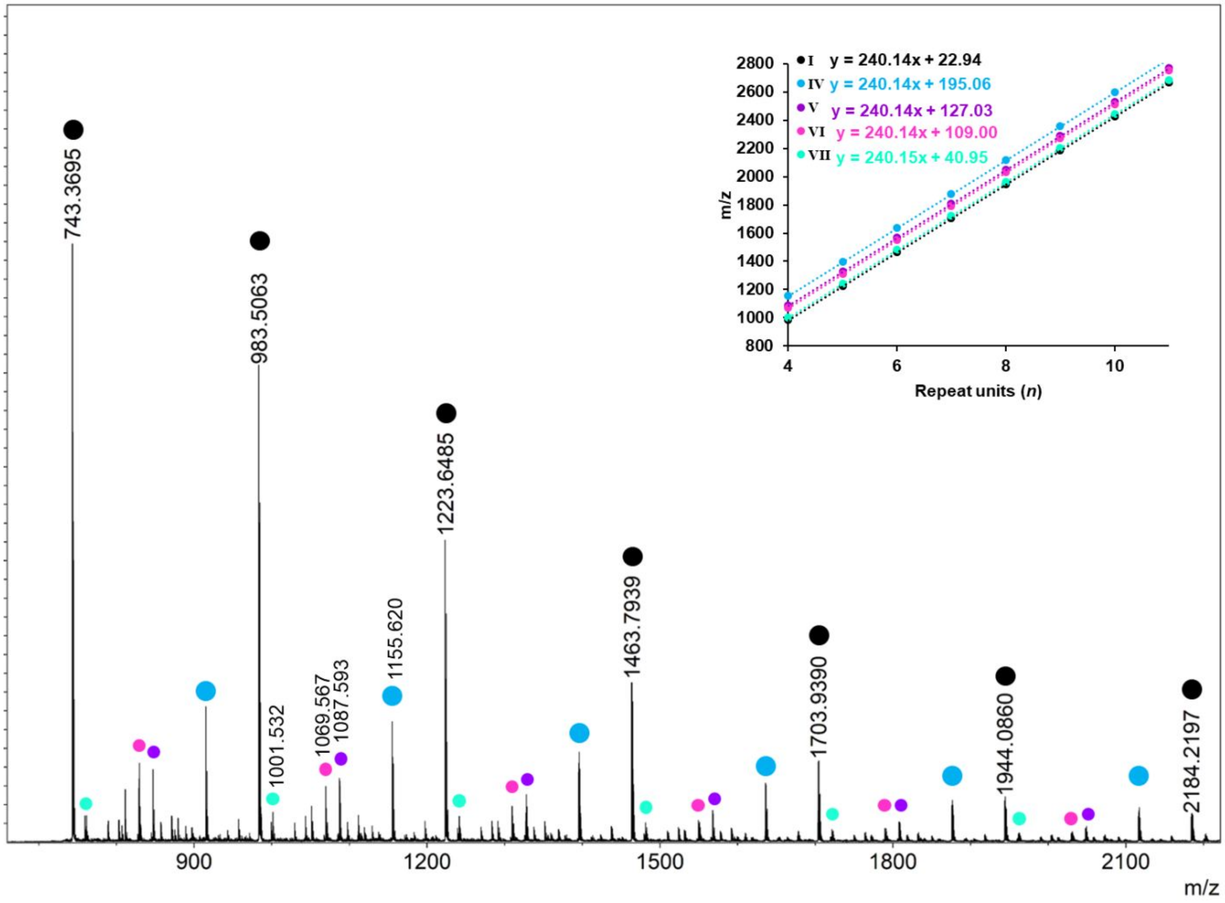

Figure S19. MALDI-TOF-MS spectrum of Poly(DC-1) after four months of ambient storage.

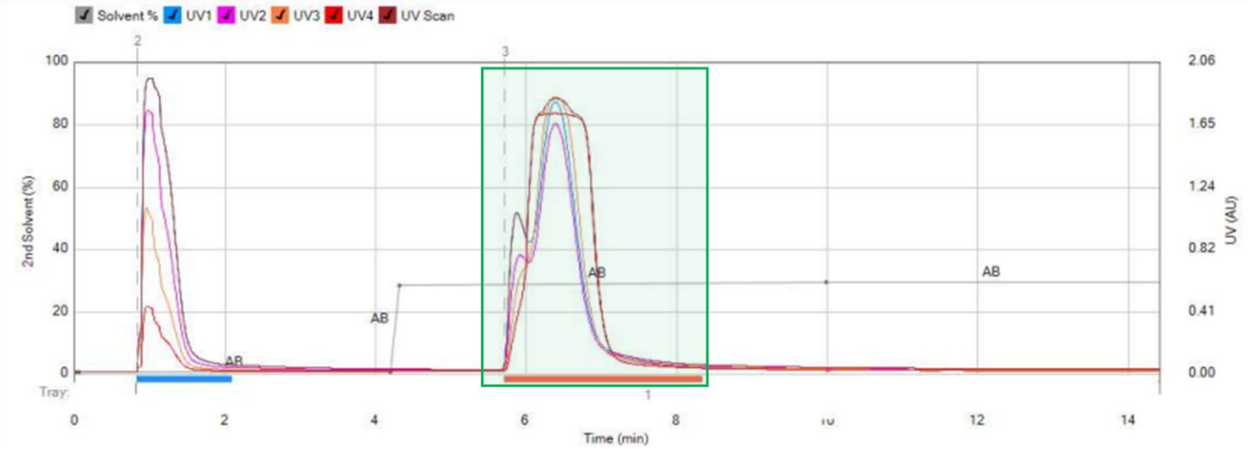

Figure S20: Elution time of DC-1 from Flash chromatography with 75:25 hexane and ethyl acetate respectively.

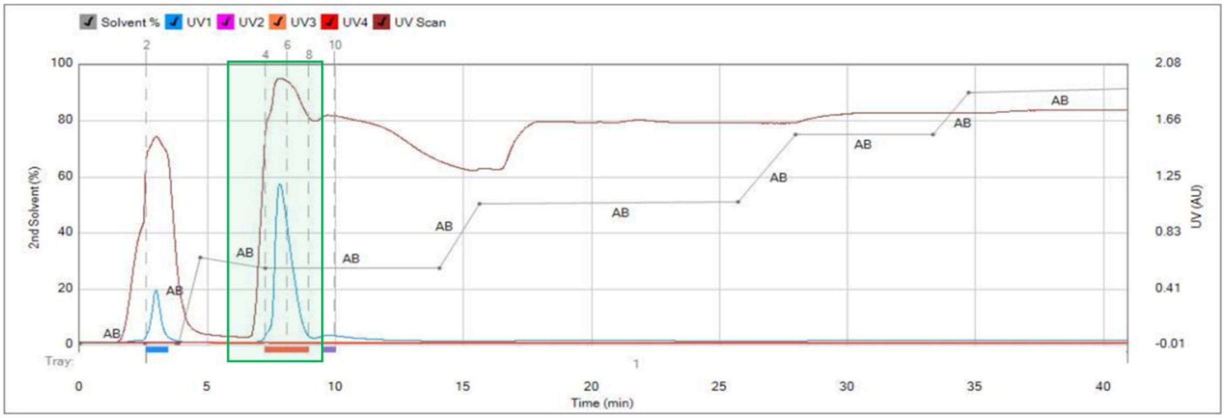

Figure S21: Elution time of DC-2 from Flash chromatography with 75:25 hexane and ethyl acetate respectively.

Preparation of 1-octadecanethiol-Grafted DC-1 and DC-2 polymers

A 50 mL round-bottom flask was charged with either DC-1 (0.4473 g, 1.98 mmol repeat units) or DC-2 (0.4470 g, 1.98 mmol repeat units) polymer, 1-octadecanethiol (0.530 g, 1.85 mmol, 0.94 equiv relative to DC-1 repeat units), and 2,2-dimethoxy-2-phenylacetophenone (DMPA, 0.0150 g, 58.5 µmol, 3.16 mol% relative to thiol), along with 35 mL of unstabilized tetrahydrofuran and a magnetic stir bar. The mixture was stirred vigorously until complete dissolution, then transferred to a UV photoreactor and irradiated at 365 nm for 8 hours. After completion, the solvent was removed under reduced pressure using a rotary evaporator. The crude product was redissolved in chloroform, cooled to 0 °C, and precipitated into diethyl ether. The precipitate was collected by vacuum filtration, washed three times with diethyl ether, and dried in a vacuum oven at 40 °C to constant weight. This procedure effectively removed residual 1-octadecanethiol to below detectable limits.

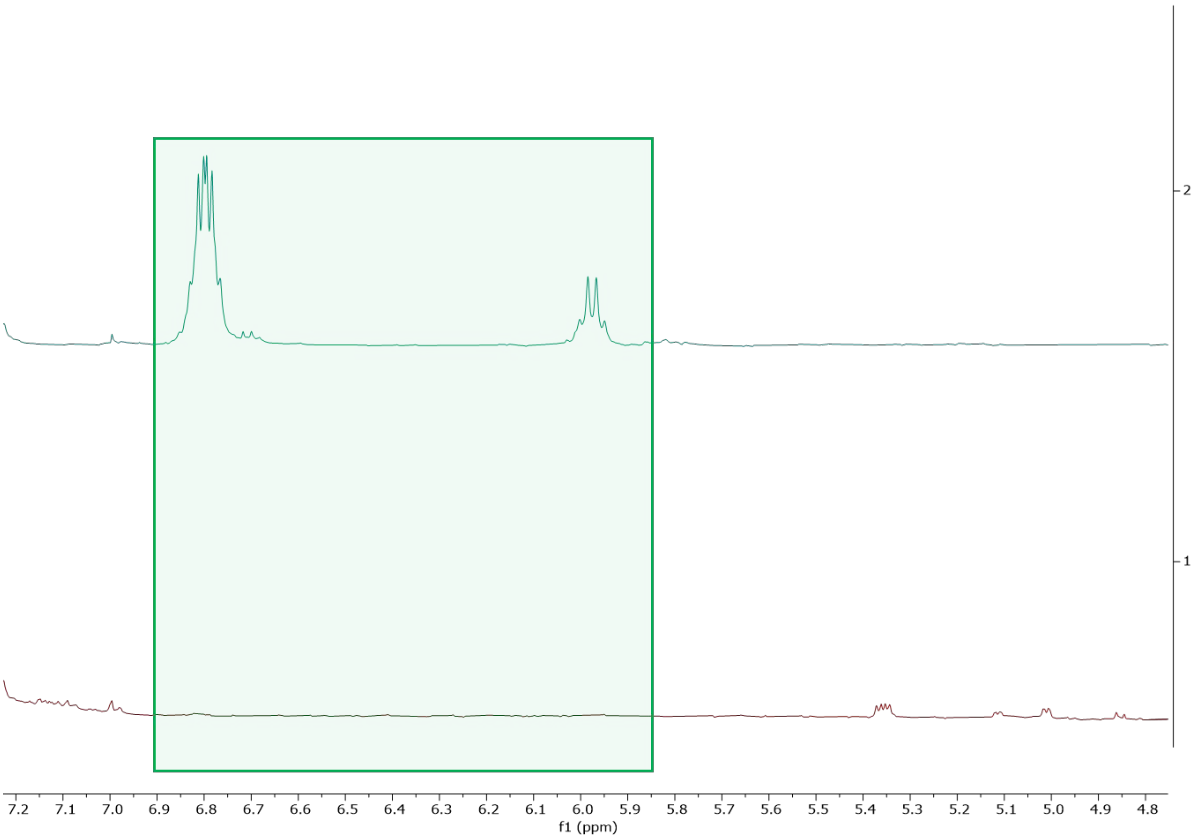

Figure S22: ^{1}H NMR ($CDCl_3$, RT) of poly(DC-1) before (top) and after (bottom) thiol-ene click reaction.

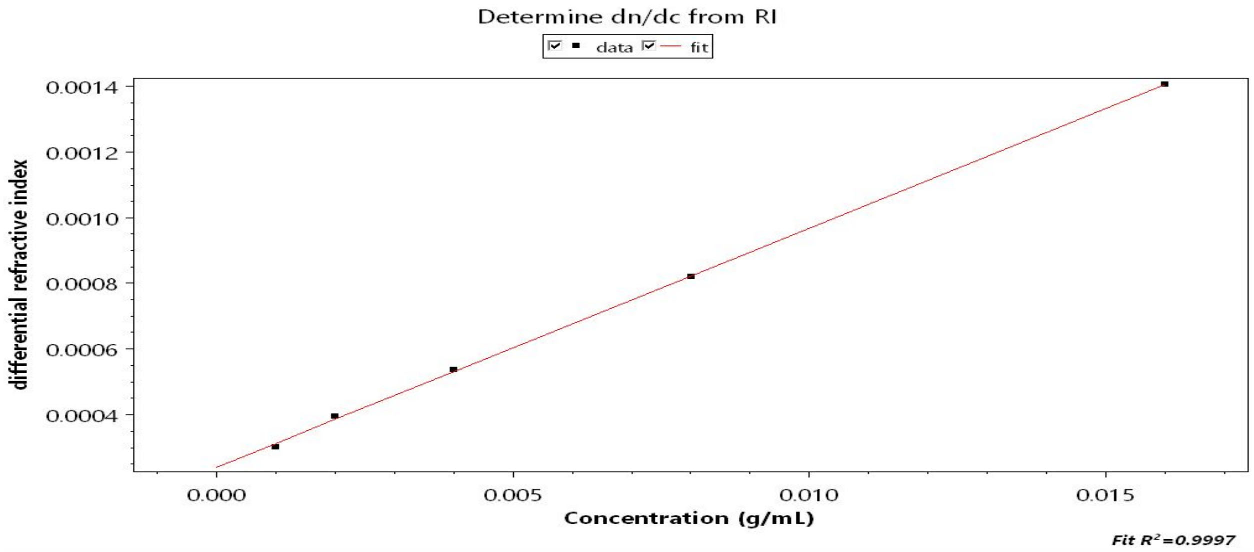

	Value
dn/dc	0.0729 ± 0.0007 mL/g

Figure S23: dRI vs concentration plot used to measure exact dn/dc value for poly (DC-1), with calculated dn/dc = 0.0729 mL/g.

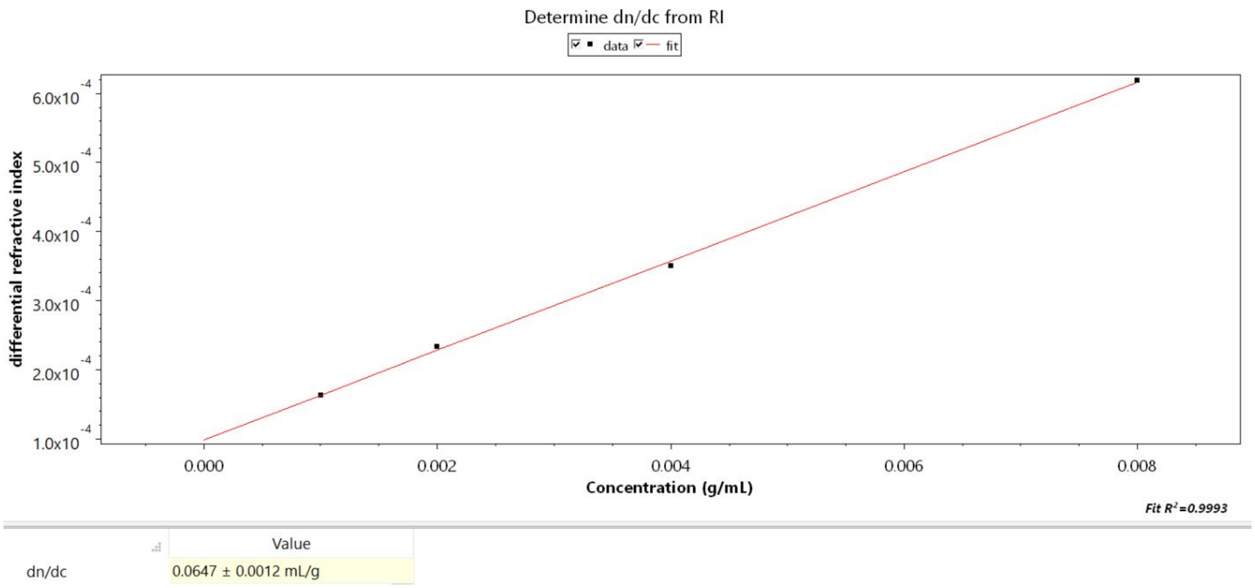

Figure S24: dRI vs concentration plot used to measure exact dn/dc value for poly (DC-2), with calculated dn/dc = 0.0647 mL/g.

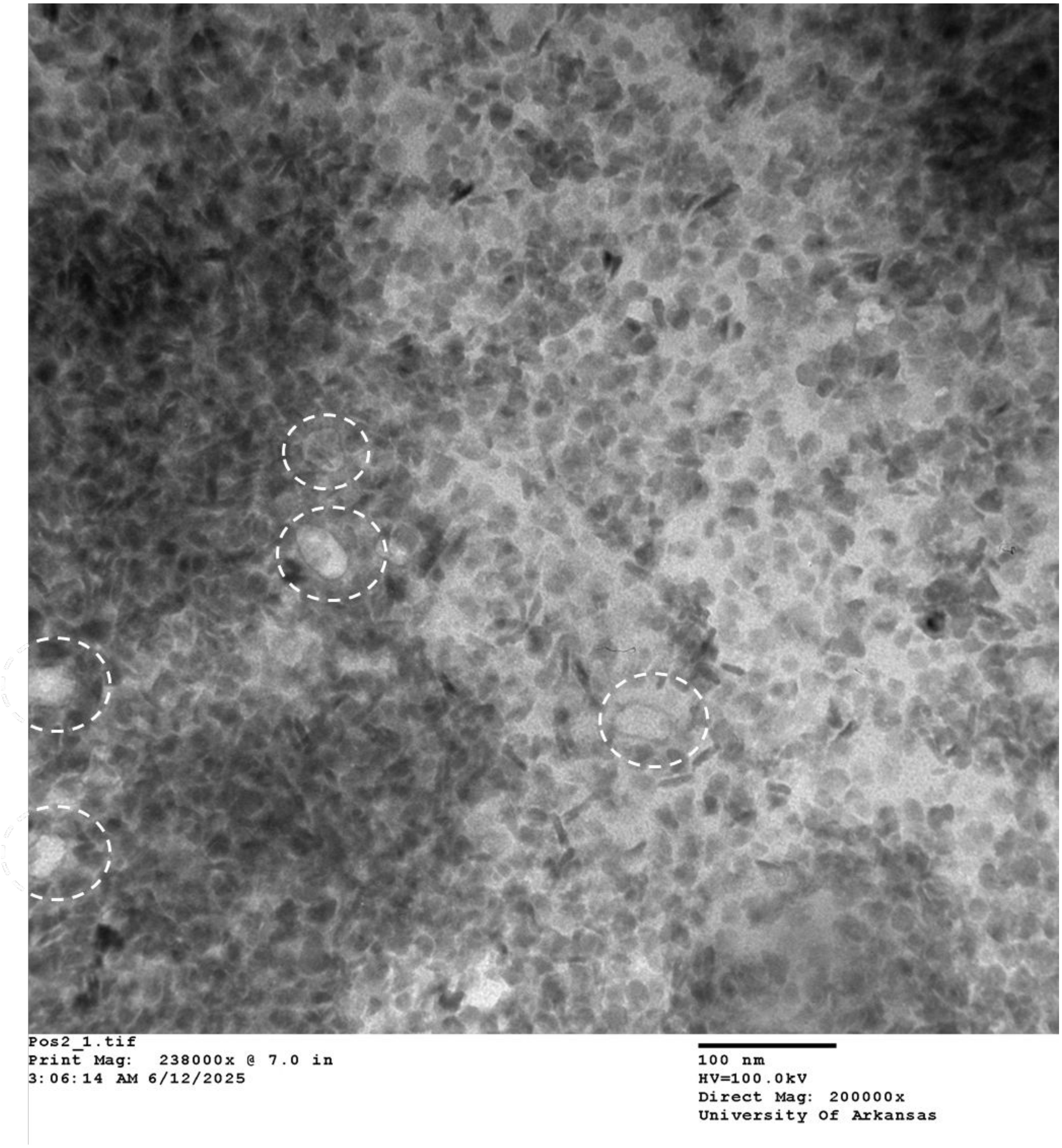

Figure S25. Transmission electron microscopy (TEM) image of 1-octadecanethiol-grafted poly(DC-1) (Table 4, run 33), labeled with guiding circles. Dark circular or spherical features are attributed to crystalline domains, while lighter hollow circular or ellipsoidal features are interpreted as individual cyclic polymer structures.

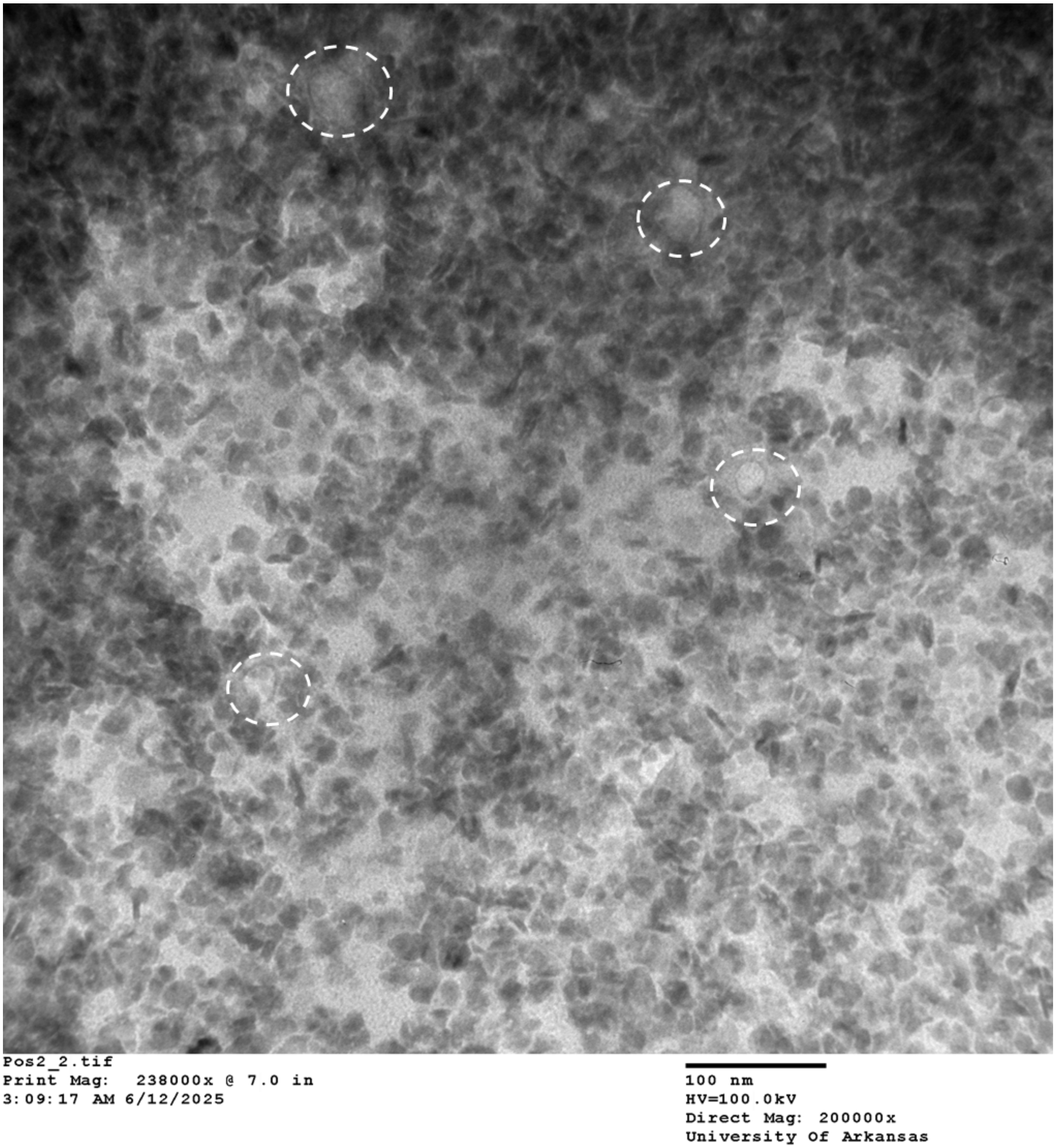

Figure S26. Additional TEM image of 1-octadecanethiol-grafted poly(DC-1) (Table 4, run 33), labeled with guiding circles. Dark circular or spherical features are attributed to crystalline domains, while lighter hollow circular or ellipsoidal features are interpreted as individual cyclic polymer structures.

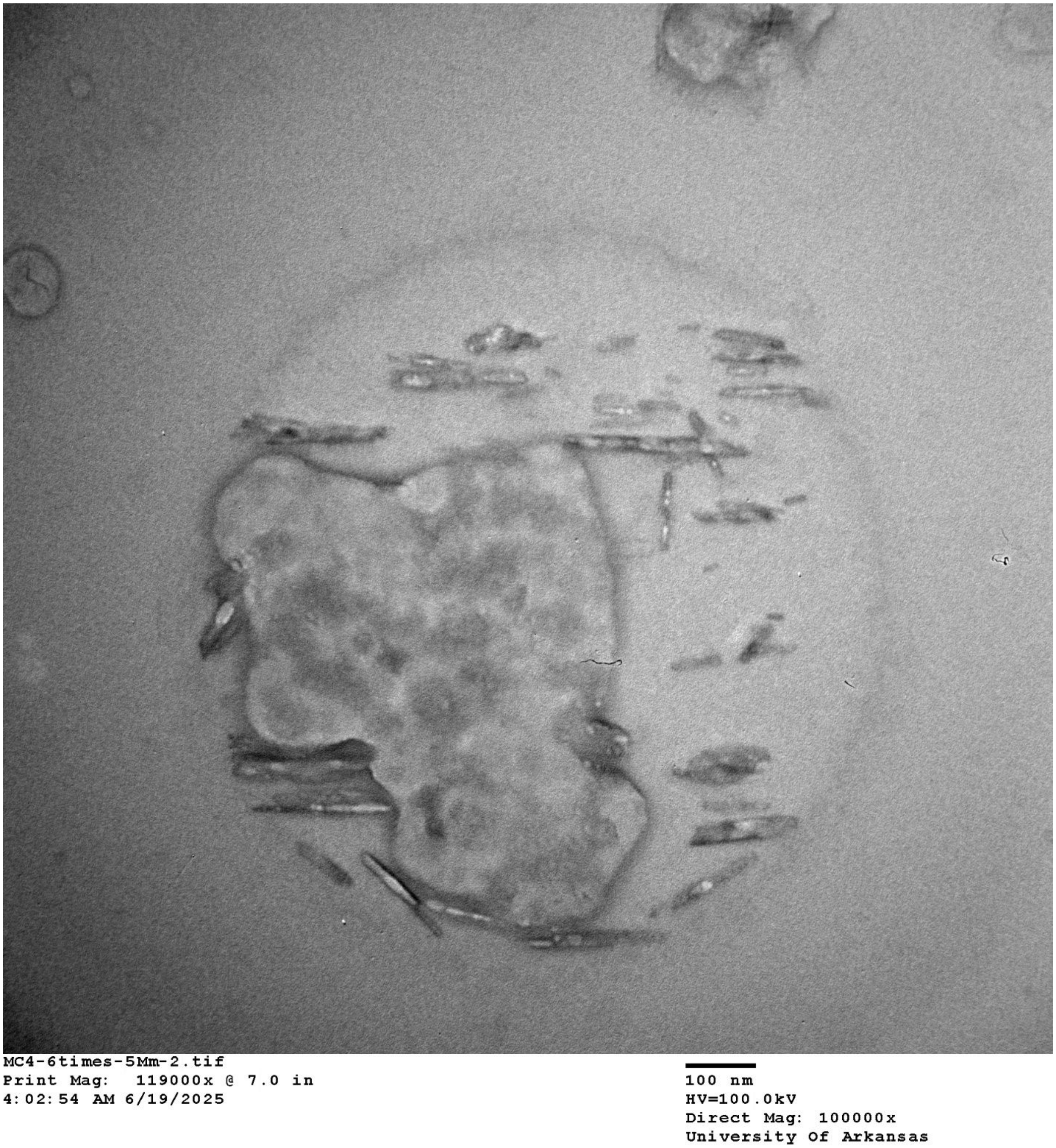

Figure S27. TEM image of 1-octadecanethiol-grafted poly(DC-1) (Table 4, run 36). Large mass of material is hypothesized to be crystalline polymer, while some smaller linear needle-like crystalline domains can be observed.
